# Sex-Dependent Differences in Blood–Urine Barrier Are Subtle but Significant in Healthy and Chronically Inflamed Mouse Bladders

**DOI:** 10.3390/ijms242216296

**Published:** 2023-11-14

**Authors:** Dominika Peskar, Mojca Kerec Kos, Uroš Cerkvenik, Alenka Nemec Svete, Andreja Erman

**Affiliations:** 1 Institute of Cell Biology, Faculty of Medicine, University of Ljubljana, 1000 Ljubljana, Slovenia; dominika.peskar@mf.uni-lj.si (D.P.); uros.cerkvenik@bf.uni-lj.si (U.C.); 2 Department of Biopharmaceutics and Pharmacokinetics, Faculty of Pharmacy, University of Ljubljana, 1000 Ljubljana, Slovenia; mojca.kerec-kos@ffa.uni-lj.si; 3 Small Animal Clinic, Veterinary Faculty, University of Ljubljana, 1000 Ljubljana, Slovenia; alenka.nemecsvete@vf.uni-lj.si

**Keywords:** bladder, blood–urine barrier, chronic cystitis, sex differences

## Abstract

The urothelium is a vital permeability barrier that prevents the uncontrolled flow of urinary components into and out of the bladder interstitium. Our study addressed the question of possible sex-specific variations in the urothelium of healthy mice and their impact on chronic bladder inflammation. We found that healthy female bladders have a less robust barrier function than male bladders, as indicated by significant differences in transepithelial electrical resistance (TEER) values. These differences could be attributed to detected higher claudin 2 mRNA expression and a less pronounced glycocalyx in females than in males. In addition, TEER measurements showed delayed barrier recovery in chronically inflamed female bladders. We found subtle differences in the expressions of genes involved in the regulation of the actin cytoskeleton between the sexes, as well as pronounced urothelial hyperplasia in females compensating for attenuated barrier function. The identified genetic variations in glycosylation pathways may also contribute to this divergence. Our findings add to the growing body of literature on the intricate sex-specific nuances of urothelial permeability function and their implications for chronic bladder inflammation. Understanding these differences could lead to tailored diagnostic and therapeutic approaches in the treatment of bladder disorders in the future.

## 1. Introduction

The urinary bladder is a hollow organ equipped for the prolonged storage of urine without the uncontrolled uptake of urine components into the bladder wall. To achieve this, the urothelium forms a highly impermeable barrier. The multilayered mammalian urothelium consists of three cell types that differ in size, location, and stage of differentiation: basal cells, intermediate cells, and superficial cells. Terminally differentiated superficial cells, called umbrella cells, are in constant contact with urine and are the key players in the transcellular and paracellular urothelial permeability barrier. Their highly specialized apical plasma membrane prevents transcellular permeability due to rigid urothelial plaques composed of uroplakins, glycosylated plasma membrane-spanning proteins [1]. Additional resistance to transcellular molecule movement is provided by cell surface glycoproteins, proteoglycans, glycosaminoglycans (GAGs), and glycolipids that form glycocalyx, a carbohydrate-enriched layer covering the apical plasma membrane of superficial urothelial cells [2,3,4], whereas paracellular urothelial permeability is regulated by numerous tight junctions between adjacent umbrella cells [5,6].

The urothelium is characterized as the tightest barrier in the body, since its transepithelial electrical resistance (TEER) ranges from 10,000 to 75,000 Ωcm^2^ under normal conditions [5,7]. However, the bladder TEER may be decreased under pathological conditions associated with injury, infection, or inflammation [8,9]. It is well documented that, upon injury, basal cells rapidly divide and gradually differentiate into intermediate cells, while intermediate cells differentiate into superficial cells, rapidly restoring the protective permeability barrier [10,11,12]. This does not appear to be the case in chronic bladder inflammation conditions, such as interstitial cystitis/bladder pain syndrome (IC/BPS). Indeed, the urothelium obtained from IC/BPS patients displays a lower differentiation status, a reduced glycocalyx, and increased permeability [9,13,14]. This loss of functional permeability barrier enables urine components to leak into the underlying lamina propria and cause symptoms such as urinary urgency and pain by sensitizing afferent nerve fibers [15,16,17].

Chronic bladder inflammatory conditions are more common in women than in men [18,19]. However, preclinical and clinical studies of bladder inflammation rarely include subjects of both sexes, and given the anatomical differences in the lower urinary tract between the sexes, the molecular basis for the sex-specific development of bladder pathology remains poorly understood [20].

We previously demonstrated sex-specific differences in the mouse bladder transcriptome following cyclophosphamide (CYP)-induced bladder inflammation [21]. In the present study, we sought to find out whether sex-dependent differences exist in the urothelial permeability barrier of healthy mice that might contribute to a greater predisposition of females to chronic bladder inflammation. In addition, we aimed to evaluate the differences in the urothelial permeability barrier between the sexes in a mouse model of chronic bladder inflammation induced by CYP. Our findings could potentially explain the high prevalence of IC/BPS in women and help to understand the differences in the severity of IC/BPS-related symptoms between the sexes.

## 2. Results

### 2.1. Healthy Bladders of Control Mice

#### 2.1.1. Urothelial Permeability Barrier Function Is Attenuated in Female Bladders Compared with Male Bladders

The measurement of TEER is a well-established method for the evaluation of cell junction integrity, which is widely used in 2D cell cultures [22] and ex vivo tissue models [23,24,25]. However, the TEER values of the bladder epithelium have never been compared between the sexes of the same animal species.

**Figure 1:**
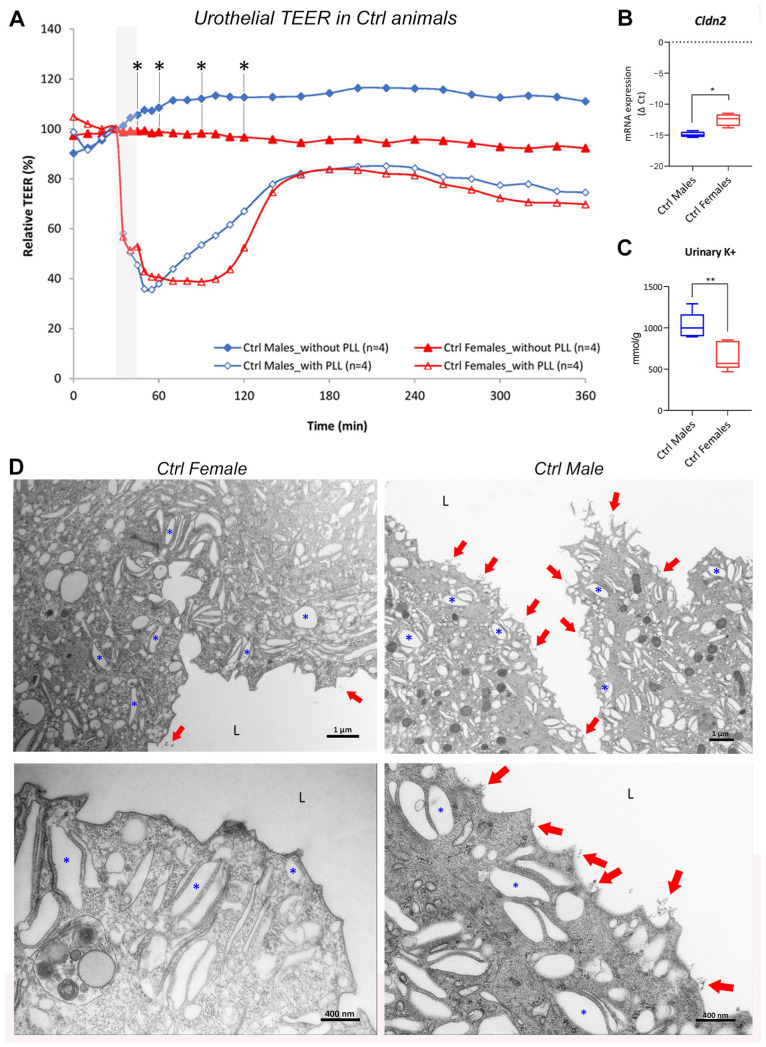
Urothelial permeability differs between the sexes of healthy mice. (**A**) Transepithelial electrical resistance (TEER) values of the urothelium show a significant difference between male and female control mice (Ctrl) at time points 45, 60, 90, and 120 min (asterisks). After the urothelium was exposed ex vivo to poly-L-lysine hydrobromide (PLL) for 15 min (shaded area), a similar drop in TEER values, followed by a similar recovery response, was observed in animals of both sexes. The graph represents the average relative TEER values of 4 animals per group. (**B**) The results of quantitative polymerase chain reaction (qPCR) analysis demonstrate a significantly higher expression of claudin 2 (*Cldn2*) in the urothelium of Ctrl females than in that of Ctrl males. The graph shows summary statistics of the negative Δ Ct determined for 4 samples per group. (**C**) The K^+^ concentration in the urine of Ctrl males is significantly higher than in that of Ctrl females. The graph represents the summary statistics of urinary K^+^ concentration normalized to urinary creatinine (mmol/g) for 5–6 samples per animal group. (**D**) Representative transmission electron microscopy (TEM) micrographs showing the glycocalyx (red arrows) on the apical plasma membrane of the superficial cells of Ctrl female and Ctrl male mice. Note that the glycocalyx is more abundant in the bladders of Ctrl males than in those of Ctrl females. Numerous fusiform vesicles (blue asterisks) in the cytoplasm indicate a high differentiation stage of the superficial cells. L—lumen of the urinary bladder. * *p* < 0.05; ** *p* < 0.01.

Our TEER measurements of healthy mouse bladders revealed differences between the sexes. Specifically, the relative TEER values were significantly lower in control (Ctrl) females than in Ctrl males at time points 45, 60, 90, and 120 min (7–16% lower in females than in males at all time points; *p* = 0.029 for each time point; Figure 1A, Supp_S4). To estimate the regenerative ability of the urothelial permeability barrier after injury, the urothelium was disrupted with poly-L-lysine hydrobromide (PLL). The measurements of TEER showed that, after the rapid drop in the TEER values of both sexes (by approximately 60% in each sex) caused by the exposure of the urothelium to PLL, the TEER values of Ctrl males began to increase rapidly and reached the maximum TEER value (85% of the initial TEER value) at 220 min. Interestingly, Ctrl female bladders stagnated at that value in the period from 60 to 110 min. After that, the TEER values started to increase rapidly, and they also reached the maximum value of TEER (85% of the initial TEER value) at 220 min.

These results indicate that the bladders of Ctrl females had a significantly higher urothelial permeability than those of Ctrl males. Additionally, the urothelial permeability barrier of Ctrl female bladders was impaired for longer than that of male bladders after acute injury, indicating a delayed recovery of the permeability barrier in Ctrl females.

#### 2.1.2. Molecular Structure and Function of Urothelial Permeability Barrier Differs between the Sexes

The passive movement of ions through the paracellular space in the urothelium is regulated by tight junctions. Claudins form the structural and functional core of tight junctions. So far, 27 members of the claudin gene family have been identified, which are generally divided into pore-forming and barrier-forming claudins [26,27]. We did not detect any differences between sexes in the expression profiles of different claudins in the whole-bladder tissue samples (based on the unsupervised clustering of RNA sequencing (RNA seq) data; Supp_S5). Similarly, a quantitative polymerase chain reaction (qPCR) analysis of urothelial tissue samples from Ctrl mice showed no significant differences in the expressions of the different claudins between the sexes (Supp_S6). However, the expression of claudin 2 (*Cldn2*) was significantly higher in the urothelium of Ctrl females than in the urothelium of Ctrl males (*p* = 0.0286; Figure 1B). Claudin 2 is a pore-forming junctional protein, and its increased expression may contribute to a greater epithelial permeability to cations (K^+^, Na^+^) but not to anions and larger molecules [28].

Urothelial barrier function is imperative for the protection of the nerve endings and muscles located in the bladder interstitium from unwanted depolarization and even cell death [17,29]. To assess the urothelial permeability to K^+^ as a key electrolyte causing the depolarization of neurons and muscle cells, urinary K^+^ concentrations were measured in Ctrl animals. Significantly lower K^+^ concentrations were found in the urine of Ctrl females than in that of Ctrl males (*p* = 0.0043; Figure 1C). This confirms the higher permeability of healthy female bladders compared to healthy male bladders, which could also be due to the higher expression of *Cldn2* in the urothelium of healthy female bladders.

#### 2.1.3. The Amount of Glycocalyx Covering the Urothelium Is Slightly Different in the Two Sexes

The glycocalyx expressed on the apical surface of umbrella cells contributes crucially to urothelial barrier function by forming a protective and anti-adherent cell surface layer for proteins, ions, and bacteria, thereby modulating the movement of charged and uncharged solutes across the urothelium [2,3]. However, the visualization of the glycocalyx in the mouse urothelium is quite difficult because it is much thinner in mice than in humans or other laboratory rodents used in studies of bladder permeability. In the present study, we observed the glycocalyx in mouse bladder samples stained with K-ferrocyanide (K_4_[Fe(CN)_6_] × 3H_2_O), which allows for components of the glycocalyx to be visualized using transmission electron microscopy (TEM) [30,31].

Using this method, we detected a difference in the amount of glycocalyx in the bladders of Ctrl males and Ctrl females. We found that the glycocalyx at the apical plasma membrane of the superficial cells at the terminal differentiation stage (indicated by the amount of fusiform vesicles in the cytoplasm) was more abundant in the bladders of Ctrl males than in those of Ctrl females (Figure 1D). This subtle difference in glycocalyx content on the urothelial surface might possibly predispose females to injury and infections and contribute to the higher bladder permeability in healthy females compared to males.

### 2.2. Chronically Inflamed Bladders of CYP-Treated Mice

#### 2.2.1. Restoration of Urothelial Permeability Barrier Function Is Impeded in Female Bladders

Urothelial dysfunction and the loss of the normal urothelial permeability barrier leading to a ‘leaky’ urothelium is one of the main symptoms of chronic bladder inflammatory conditions [3,13].

**Figure 2:**
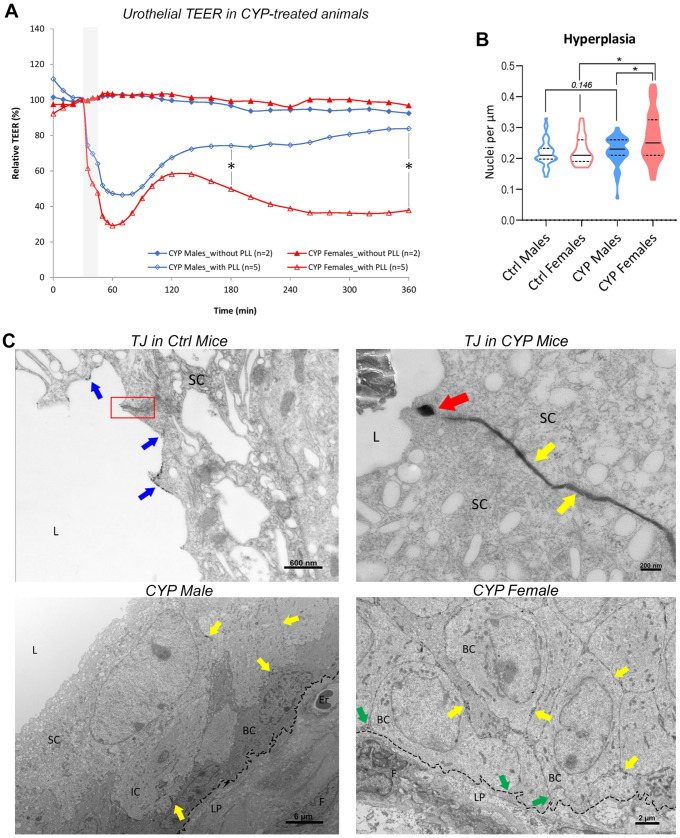
Urothelial barrier function is impaired in female mice with chronic bladder inflammation. (**A**) Urothelial transepithelial electrical resistance (TEER) values in cyclophosphamide (CYP)-treated mice show a greater drop in females than in males after ex vivo exposure to PLL (shaded area) and a significant difference in recovery response at time points 180 and 360 min (asterisks). The graph shows the average relative TEER values of 2–5 animals per group. (**B**) Urothelial hyperplasia is significantly more pronounced in female CYP-treated mice than in male CYP-treated mice. The graph represents the data distribution and summary statistics of the number of nuclei per 1 µm urothelial length obtained from 5 regions of interest in 4–5 animals per group. * *p* < 0.05. (**C**) Representative transmission electron microscopy (TEM) micrographs of the bladder tissue labeled with an electron-dense LaNO_3_ tracer. In Ctrl animals of both sexes, the integrity of the tight junctions (TJs; red box) is maintained; therefore, LaNO_3_ (blue arrows) is only observed on the apical surface of the superficial cells (SCs). In CYP-treated males and females, the integrity of TJ is compromised (red arrow), enabling the penetration of LaNO_3_ from the apical surface of the superficial cells into the intercellular spaces of the urothelium (yellow arrows). However, only in CYP-treated females did LaNO_3_ reach the basal lamina (dotted line) at several sites (green arrows), while in CYP-treated male mice, it reached the basal lamina only occasionally. L—lumen of the urinary bladder; IC—intermediate cell; BC—basal cell; LP—lamina propria; F—fibroblast; Er—erythrocyte.

In order to assess the possible differences in urothelial barrier functionality between the sexes following the induction of chronic inflammation, we performed TEER measurements on the bladders of CYP-treated male and female animals. Interestingly, our results showed no distinct decrease in the relative TEER values of the CYP-treated animals compared to those of the controls (Supp_S7), indicating a similar level of permeability barrier recovery after the repeated administration of CYP in both sexes. However, after ex vivo disruption of the urothelium with PLL, the responses of the urothelium of males and females differed drastically (Figure 2A). Initially, the drop in the relative TEER values after exposure to PLL was higher in females (by 71%) than in males at the 60 min time point (by 53%; *p* = 0.063; Supp_S4). Later, at the beginning of the restoration phase (at the 120 min time point), the relative TEER values reached 67% of the initial TEER value in males and continued to increase over time, while the TEER values in females reached 58% of the initial TEER value at the same time point and only decreased thereafter (Figure 2A). At 180 and 360 min, the relative TEER values were significantly lower in females than in males (49% and 37% of the initial TEER value in females, 74% and 83% of the initial TEER value in males; *p* = 0.032 for both comparisons; Figure 2A; Supp_S4).

These results suggest that female bladders with a pre-damaged urothelium due to CYP are more sensitive to further injury and fail to recover. We can therefore speculate that, in chronic aseptic cystitis, severely attenuated recovery of the urothelial permeability barrier may lead to repeated injury of the bladder mucosa, further reinforcing the vicious cycle of chronic inflammation.

In line with this hypothesis, we aimed to identify possible differences in the components of the urothelial permeability barrier between the sexes that might lead to the progression of increased permeability during the duration of chronic inflammation. In particular, we focused on the regeneration of urothelial tissue after injury, which includes cell proliferation, the re-establishment of functional cell junctions, and the restoration of the glycocalyx layer.

#### 2.2.2. Urothelial Hyperplasia Is More Prominent in Female Bladders

Under physiological conditions, the urothelium is a quiescent epithelium with a turnover rate of 40 weeks in adult rodents [32]. The slow turnover rate is crucial for the urothelium to function as a constant permeability barrier [33]. However, the urothelium starts to be highly proliferative immediately after injury [10,11,12].

In the present study, urothelial hyperplasia was detected in the bladders of CYP-treated mice. We therefore quantified the hyperplasia by determining the density of the urothelial nuclei. Nuclear density was significantly higher in CYP-treated female bladders than in CYP-treated male bladders (*p* = 0.0182, Figure 2B), although both sexes had an increased number of nuclei compared to the corresponding controls.

We can speculate that the attenuated permeability barrier in CYP-treated female bladders is compensated for by an increased number of urothelial cells.

#### 2.2.3. Subtle Differences in Urothelial Cell Junction-Actin Cytoskeleton Network Are Present between the Sexes

Well-established and functioning cell junctions, especially the tight junctions, are crucial for maintaining the active blood–urine barrier and preventing the paracellular passage of urinary components into the bladder interstitium. To assess the integrity of tight junctions in the bladders of both sexes after treatment with CYP, bladder tissue samples were incubated in lanthanum nitrate (LaNO_3_), an electron-dense compound. A TEM analysis showed disrupted intercellular junctions and enlarged intercellular spaces between the urothelial cells in the urothelium of both sexes. However, in CYP-treated females, LaNO_3_ penetration was observed in the paracellular spaces of the entire urothelium reaching the basal lamina at several sites. In contrast, LaNO_3_ rarely reached the basal lamina in the bladders of CYP-treated males (Figure 2C). In the bladders of control mice, where the tight junctions between the superficial cells were maintained, LaNO_3_ did not penetrate the tight junctions and was therefore only observed at the apical plasma membrane of the umbrella cells. These results point to higher paracellular epithelial permeability in the bladders of CYP-treated females than in those of CYP-treated males.

**Figure 3:**
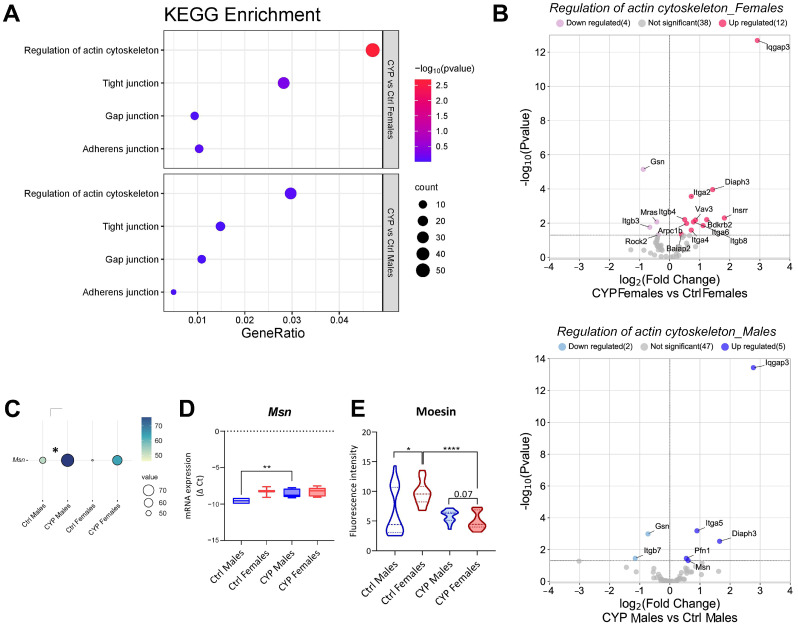
Subtle differences between the sexes in the regulation of cell junction–actin cytoskeleton network in mice with chronic bladder inflammation. (**A**) Kyoto Encyclopedia of Genes and Genomes (KEGG) enrichment analysis shows greater enrichment of cell junction-related pathways in females than in males after treatment with CYP. The regulation of actin cytoskeleton is significantly more enriched in females versus males (*p* = 0.00205). A higher *p*-value is indicated by the red color, and a higher number of genes in each pathway is indicated by the size of the bubble. (**B**) Volcano plots show the subtle differences in the expression of genes involved in the regulation of the actin cytoskeleton between the sexes. The names of significantly deregulated genes (*p*_adj_ < 0.05) are displayed. (**C**) The balloon plot represents the difference in average Fragments Per Kilobase Million (FPKM) values of *Msn* expression between Ctrl males, CYP males, Ctrl females, and CYP females as determined by RNA seq on 3 samples per group. *Msn* expression is significantly increased in males after CYP treatment. A higher value is indicated by a darker blue and a larger circle. (**D**) Quantitative polymerase chain reaction (qPCR) validation of *Msn* expression in urothelial tissue samples confirming a significant increase in *Msn* in males after CYP treatment. The graph shows summary statistics of negative Δ Ct determined for 3–6 samples per group. (**E**) Moesin expression in the urothelium based on quantification of fluorescence intensity. The graph shows data distribution and summary statistics of fluorescence intensity obtained in 5 regions of interest with 3 animals per group. * *p* < 0.05; ** *p* < 0.01; **** *p* < 0.0001.

The Kyoto Encyclopedia of Genes and Genomes (KEGG) analysis obtained from our previously published RNA seq data [21] showed minimal differences between the sexes in the enrichment of cell junction-related pathways (‘Tight junctions’, ‘Adherens junctions’, ‘Gap junctions’) after treatment with CYP. However, as shown in Figure 3A, all processes were more enriched (indicated by bubble color) and contained a higher number of genes (indicated by bubble size) in females than in males. The results of the RNA seq data analysis also showed that CYP treatment had a significant impact on the expression profile of cell junction-related genes (Supp_S8), but the subtle differences between the sexes were evident only in the number and uniqueness of significantly deregulated genes (*p*_adj_ < 0.05; three up and three down in females; five up and one down in males; four out of six genes were uniquely deregulated in each sex; Supp_S9).

However, the qPCR analysis of cell junction-related genes (*Tjp1*, *Cldn1*, *Cldn4*, *Cldn5*, *Cldn10*, *Cldn2*, *Ctnna1*, *Ctnnb1*, and *Gja1*) performed on the urothelial tissue samples failed to show any significant sex-specific changes in gene expression after treatment with CYP (Supp_S6). These data indicate that the sex-specific changes in the expressions of junctional proteins, if present, are far too subtle to be detected in a small sample size such as ours.

The actin cytoskeleton is an important component of epithelial barrier function. It is associated with the plasma membrane, and tight- and adherens junction proteins, which together form a junctional complex between adjacent urothelial cells and play an important role in transmitting regulatory signals for paracellular barrier control [6]. Our KEGG analysis revealed a significant enrichment of the ‘Regulation of actin cytoskeleton’ pathway in CYP-treated female mice (*p* = 0.00205; Figure 3A). Based on independent clustering, treatment with CYP had a significant impact on the expression profiles of the genes involved in this pathway, but no distinctive differences between the sexes were detected (Supp_S10). However, the expression profiles of the significantly deregulated genes (*p*_adj_ < 0.05) in the aforementioned pathway differed between the sexes, as indicated by their number and uniqueness in each sex (of 20 significantly deregulated genes, 12 were upregulated and 4 were downregulated in females, while 5 were upregulated and 2 were downregulated in males; Figure 3B).

Moesin, as one of the actin-binding proteins, influences the organization of the actin cytoskeleton and the maintenance of epithelial integrity by regulating cell polarity [34]. Our RNA seq data showed that the moesin gene *Msn* was significantly upregulated in males after treatment with CYP (Figure 3C). The significant upregulation of *Msn* mRNA was also confirmed via a qPCR validation of urothelial tissue samples from CYP-treated males (*p* = 0.0095; Figure 3D), while no differences were found in CYP-treated females. The quantification of urothelial moesin protein expression showed a significant reduction in moesin expression in females after treatment (*p* < 0.0001, Figure 3E), while the reduction was not significant in males. A trend of lower moesin expression in CYP-treated females than in CYP-treated males was also observed. Interestingly, the moesin expression was significantly higher in Ctrl males than in Ctrl females (*p* = 0.0256, Figure 3E).

Based on the increased expression of moesin mRNA in the urothelium of treated males and the significant decrease in the expression of moesin protein in the urothelium of treated females, we can hypothesize that the organization of the actin cytoskeleton in urothelial cells might be more compromised in the injured urothelium of females than in that of males, possibly contributing to the decreased integrity of cell junctions and, consequently, a less efficient urothelial barrier in females than in males.

#### 2.2.4. Urothelial Glycosylation Pattern Differs between the Sexes

The glycosylation profile of urothelial cells is altered in a chronically inflamed bladder, and a loss of the protective GAG layer may contribute significantly to an increased permeability of the urothelium [3,35].

**Figure 4:**
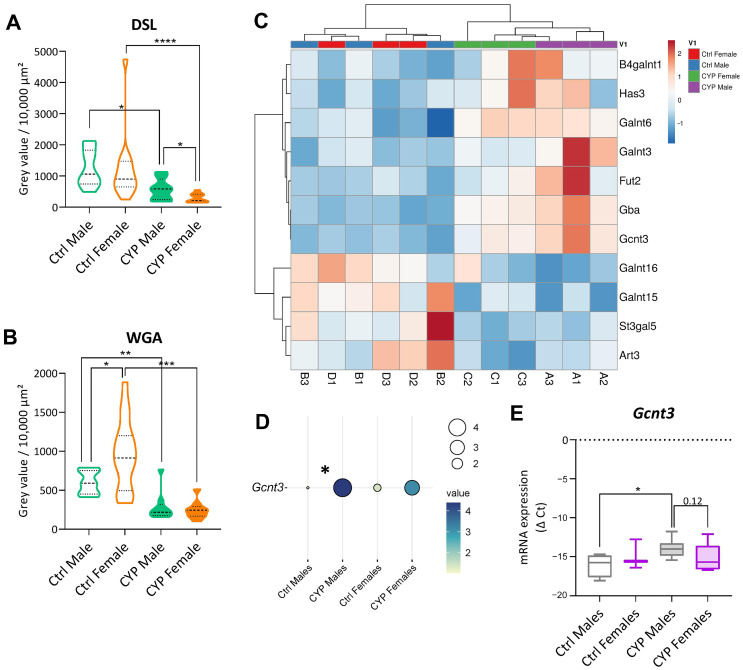
Differences in urothelial glycosylation of chronically inflamed bladders are sex-specific. (**A**,**B**) The binding intensity of the *Datura stramonium* lectin (DSL) and Wheat Germ agglutinin (WGA) to the urothelium of mice of both sexes is significantly reduced after CYP treatment compared to Ctrl mice, and it is more diminished in females than in males. The graphs show the data distribution and summary statistics of gray values/10,000 µm^2^ obtained from 4 regions of interest with 5 animals per group. (**C**) Heatmap shows independent clustering of significantly deregulated (p_adj_ < 0.05) glycosyltransferase genes (n = 11) detected in both sexes after CYP treatment using RNA seq with 3 animals per group. Ctrl females (red, D1–3), Ctrl males (blue, B1–3), CYP females (green, C1–3), and CYP males (purple, A1–3) are compared. The rows and columns are clustered based on correlation distance and average linkage. Lower and higher gene expression is indicated by blue and red color, respectively. (**D**) The balloon plot represents the difference in average Fragments Per Kilobase Million (FPKM) values of *Gcnt3* expression between Ctrl males, CYP males, Ctrl females, and CYP females obtained using RNA seq on 3 animals per group. *Gcnt3* expression is significantly increased in males after CYP treatment. The higher value is indicated by a darker blue and a larger circle. (**E**) Quantitative polymerase chain reaction (qPCR) validation of *Gcnt3* expression in urothelial tissue samples confirms a significant increase in *Gcnt3* expression in CYP-treated males compared to Ctrl males. The graph shows summary statistics of negative Δ Ct determined for 3–6 samples per animal group. * *p* < 0.05; ** *p* < 0.01; *** *p* < 0.001; **** *p* < 0.0001.

In the present study, we performed lectin histochemistry for the lectins Concavalin A (ConA), *Datura stramonium* lectin (DSL), Jacalin, and Wheat Germ agglutinin (WGA) to identify any differences in urothelial glycosylation after treatment with CYP between the sexes. We found that the intensity of lectin binding to the urothelium was significantly diminished in the CYP-treated animals of both sexes compared to the controls for all lectins analyzed (Figure 4A,B; Supp_S11). Furthermore, the fluorescence intensity of DSL labeling in the urothelium of CYP-treated females was significantly lower than in that of CYP-treated males (*p* = 0.036, Figure 4A; Supp_S12). Similarly, the decrease in fluorescence intensity of WGA labeling was more significant in CYP-treated females than in CYP-treated males (*p* < 0.0001 for females; *p* = 0.0037 for males; Figure 4A; Supp_S12). These data demonstrate sex-specific changes in the glycosylation profile of urothelial cells in bladders with CYP-induced cystitis.

The processes of protein glycosylation and the formation of oligosaccharide chains are carried out by the processing enzymes glycosidases and glycosyltransferases. Glycosyltransferases are enzymes responsible for the posttranslational modification that takes place in the endoplasmic reticulum and the Golgi apparatus. The differences in the glycoprotein expression pattern of the urothelium between the sexes could be attributed to the differential expression of glycosyltransferases.

We used the Human Protein Atlas database (available at https://www.proteinatlas.org/ (accessed on 11 July 2023)) to create a gene set of glycosyltransferases (Supp_S13), and RNA seq data for the gene set were extracted. As demonstrated by the unsupervised clustering analysis, CYP treatment had no significant effect on the expressions of glycosyltransferases in the mouse bladders (Supp_S14). However, the analysis resulted in the distinctive clustering of CYP-treated animals by sex based on the expression of 11 significantly deregulated glycosyltransferase genes (*p*_adj_ < 0.05). These results reveal that some differences in bladder glycosyltransferase expression exist between the sexes in chronic inflammation, mainly as higher expression of these genes in the bladders of CYP-treated males compared to CYP-treated females (Figure 4B). At the same time, the clustering showed no obvious difference in glycosyltransferase expression between control animals.

Mucin-type glucosaminyl (N-Acetyl) transferase 3 (C2GnT-M), encoded by the *Gcnt3* gene, was significantly upregulated only in males after CYP treatment based on the RNA seq data (Figure 4D). Its sex-specific upregulation was additionally confirmed via a qPCR of urothelial tissue samples (*p* = 0.0381, Figure 4D). A trend towards a higher expression of *Gcnt3* was also observed in CYP males than in CYP females (*p* = 0.12), indicating higher activity of the C2GnT-M enzyme in males after CYP treatment. C2GnT-M is responsible for the formation of core 2 and core 4 in mucin-type O-glycans [36] and, thus, influences the extensions of the O-glycosylation branches. Since mucins are highly glycosylated proteins that play a central role in maintaining epithelial homeostasis by providing additional protection against the extracellular environment [37], a *Gcnt3*-dependent reduced length of carbohydrate branches protruding into the bladder lumen could contribute to an impaired barrier of the female urothelium.

## 3. Discussion

Sex differences in the inflammatory response in humans are well established [38]. In urinary tract infections, sex hormones influence the extent of bacterial infestation and the successfulness of its resolution [39]. However, the question of why an aseptic bladder inflammation condition such as IC/BPS affects women more often than men remains unanswered. In the search for the reasons for this difference in prevalence, the sex-dependent distinction in urothelial permeability barrier has never been addressed.

In the present study, we aimed to elucidate the sex-dependent differences in the urothelial permeability barrier of healthy C57BL/6J mice, which are commonly used as model animals for various lower urinary tract disorders, including cystitis. In addition, we analyzed some sex-dependent differences in the structural and functional recovery of the urothelial permeability barrier after the induction of aseptic chronic cystitis using a mouse model treated with multiple injections of CYP.

In healthy mice, we found that the urothelial barrier was more permeable in females than in males, as shown by significantly lower TEER. The barrier was also impaired for longer in females than in males after the acute injury induced by PLL treatment ex vivo. To the best of our knowledge, the differences in healthy urothelial barrier function between the sexes have never been studied before. One of the possible reasons for the increased urothelial permeability in females could be the different molecular structure of the urothelial barrier. Our results provide evidence for a significantly higher expression of claudin 2 (*Cldn2*) in females than in males. Claudin 2 is a pore-forming junctional protein that is cation-selective [16,28,40]. Its overexpression may contribute to the development of bladder inflammation via increased urothelial permeability for K^+^ ions [16,17,29]. In the present study, we showed that urinary K^+^ concentrations were significantly lower in Ctrl females than in Ctrl males, indicating the loss of urinary K^+^ through claudin 2-mediated leakage across the urothelium. However, it cannot be completely excluded that the sex-dependent expression of electrolyte transporters in the normal mouse renal epithelium contributes to the discrepancies in urinary K^+^ concentrations between the sexes [41].

The protective glycocalyx in the urinary bladder consists of cell surface proteoglycans, glycoproteins, and glycolipids associated with the apical plasma membrane of superficial cells [2,3]. In the present study, we used K-ferrocyanide labeling to visualize the otherwise difficult-to-detect glycocalyx in the urothelium of healthy mice. We showed that the glycocalyx on the apical surface of terminally differentiated umbrella cells is more abundant in male than in female bladders. We hypothesize that this subtle difference in the content of the protective glycocalyx layer may be one of the predispositions of females for attenuated urothelial barrier function.

It is well known that urothelial barrier function is diminished in chronic bladder inflammation conditions, characterized by the loss of the protective mucus layer and increased paracellular permeability, often due to a lower differentiation status of urothelial cells [3,14]. Interestingly, we found that CYP treatment did not cause a significant decrease in the relative TEER values of animals of either sex, which is in line with the findings of Chelsky and coworkers [42]. However, following acute injury to the urothelium induced by PLL ex vivo, the recovery of barrier function was slower in female than in male bladders. As mentioned earlier, the urothelium of healthy female mice takes longer than that of males to regenerate after PLL-induced acute injury. This delay in barrier regeneration causes a prolonged exposure of lamina propria with its nerve endings and blood vessels to the external environment, leading to more severe damage of the bladder wall. After repeated injury, the urothelium of female mice with a lower regenerative capacity fails to recover over time, as observed in the TEER measurements of CYP-treated bladders. Therefore, we speculate that, in chronic aseptic cystitis, the attenuated recovery of urothelial barrier reinforces the vicious cycle of chronic inflammation in female patients.

In search of possible mechanisms contributing to the sex-dependent differences in bladder barrier re-establishment after injury, we found that the urothelial hyperplasia was more pronounced in female mice than in male mice. Hyperplasia is a normal finding in a CYP-induced mouse model of bladder inflammation. Boudes and colleagues observed it in male C57BL/6 mice [43], while Golubeva and colleagues confirmed it in female C57BL/6 mice [44]. However, our study is the first to point out the differences in the extent of hyperplasia between the sexes. We speculate that the increased number of urothelial cells in the female urothelium is a compensatory mechanism for the attenuated permeability barrier after CYP-induced injury.

The extent of hyperplasia appeared to be correlated with the paracellular permeability of the urothelium. As the TEM analysis revealed, LaNO_3_ permeated the paracellular spaces of the urothelium of CYP females to a greater extent than in CYP males, suggesting higher paracellular epithelial permeability in the bladders of CYP-treated females than in those of CYP-treated males. However, we did not detect any significant effect of CYP treatment on the expression of the various cell junction proteins, a finding similar to that of Golubeva and colleagues [44].

The KEGG analysis showed a significant enrichment in the ‘Regulation of actin cytoskeleton’ pathway in female mice after CYP treatment, indicating a higher number of significantly deregulated genes in females than in males. This could be a consequence of a higher cell number due to comprehensive hyperplasia in females. The circumferential organization of the actin cytoskeleton is characteristic of basal and intermediate urothelial cells, as well as of poorly differentiated superficial cells that dominate in the urothelium after injury [45], thereby adding to the overall content of actin cytoskeleton elements in the female urothelium. However, the importance of functional actin organization in the maintenance of epithelial integrity is not negligible [6]. The expression of the moesin gene *Msn* was higher in the urothelial samples from CYP-treated males compared to in those from CYP-treated females, while the protein expression was lower in the urothelium of CYP-treated females compared to CYP-treated males. The discrepancy between mRNA and protein expressions might be due to the microRNA-regulated inhibition of *Msn* mRNA translation, possibly by miR-145-5p, which was found to be upregulated in the bladders of CYP-treated animals (our unpublished data). Moesin is a member of the ERM complex (the ezrin, radixin, and moesin complex) and a key organizer of specialized membrane domains by interacting with transmembrane proteins and the cytoskeleton, among others [46]. Moesin is locally and functionally associated with the formation of adherens junctions by stabilizing them in a Rho GTPase/F-actin-dependent manner [34,47]. Although moesin is required for the maintenance of epithelial integrity and the appropriate organization of the actin cytoskeleton, its depletion has been shown not to completely change the stability of adherens junctions [34]. This finding is consistent with the results of our study, where no significant differences in TEER and the expression of genes related to cell junctions were found in treated animals. However, we assume that the decreased expression of moesin in CYP females compared to in CYP males might contribute to a higher paracellular permeability of the urothelium.

Chronic inflammatory conditions can significantly affect the glycosylation profile of urothelial cells, contributing to impaired barrier function [3,35]. The importance of restoring a deficient urothelial glycocalyx is also reflected in the use of glycosaminoglycan replenishment as one of the most common and successful therapy options used for IC/BPS [48]. Our analysis of urothelial glycosylation revealed a significant decrease in the intensity of lectin binding as a consequence of CYP treatment. Significant differences in DSL and WGA binding between male and female mice additionally indicated a sex-specific urothelial response to chronic inflammation.

Protein glycosylation is highly influenced by the expressions of glycosyltransferase enzymes, which we found to differ between CYP males and CYP females. The significant increase in the expression of *Gcnt3* was confirmed in the urothelial samples from CYP males, indicating a higher activity of the C2GnT-M enzyme, which may contribute to the extent of the O-glycosylation of mucins present in the urothelium. Mucins contribute significantly to the urothelial barrier and anti-adhesive function by protruding from the epithelial luminal surface with their extensive, rigid, and highly glycosylated extracellular domain [37,49]. It is worth noting that the rigidity of this protective layer can often reduce the effectiveness of cancer therapy. Therefore, the inhibition of the *Gcnt3* gene has been proposed for improving the effect of chemotherapy by reducing the O-glycosylation of mucins in breast cancer [50]. The higher expression of *Gcnt3* in the urothelium of male mice therefore suggests a more complex glycoprotein structure that could contribute to a more successful restoration of barrier function after CYP treatment.

In conclusion, the results of our study demonstrate for the first time that subtle differences exist between the sexes in the integrity and restorative capacity of the urothelium as a permeability barrier. However, we speculate that these differences, when they accumulate and interact, contribute significantly to differences in the development of bladder diseases such as IC/BPS in both sexes. They should therefore be considered in diagnostic and therapeutic approaches to aseptic chronic bladder inflammation. The results of this study provide a conceptual framework that should be additionally considered in future research on sex-dependent bladder pathology.

## 4. Materials and Methods

### 4.1. Animals and Induction of Chronic Bladder Inflammation

Animal experiments were performed in accordance with the Administration of the Republic of Slovenia for Food Safety, Veterinary Sector, and Plant Protection, permit number U34401-4/2020/10. A total of 44 adult (12–14 weeks old) C57BL/6J mice (Jackson Laboratory, Bar Harbor, ME, USA) of both sexes (22 males and 22 females) weighing 20–30 g were included in the study. The animals were housed in polycarbonate cages in groups of five at a constant humidity (55%) and temperature (22 °C) and a 12/12 h light cycle. Throughout the experiment, the animals had ad libitum access to food and drinking water. Before the start of the experiment, the mice were allowed an acclimatization period of 14 days. During this period, the mice were randomized into the control group (Ctrl mice, n = 20) and the cyclophosphamide-treated group (CYP mice, n = 24). In the CYP group of mice, chronic aseptic cystitis was induced utilizing 4 i.p. injections of CYP (Sigma-Aldrich Chemie, Steinheim, Germany; 80 mg/kg diluted in sterile saline) on days 0, 2, 4, and 6 of the 8-day experiment, according to a previously established method [43,44]. The Ctrl group received equivalent volumes of sterile saline. The animals were weighed daily and monitored for the presence of discomfort or pain. The animals were euthanized on day 8 of the experiment via CO_2_ asphyxia in a mouse euthanasia chamber. Urinary bladders were excised and processed for further analysis, as described below.

### 4.2. Ex Vivo Measurements of Urothelial Transepithelial Electrical Resistance

TEER measuring both transcellular and paracellular transport was measured ex vivo using side-by-side diffusion chambers (Ussing chambers). After euthanasia, the mouse urinary bladders were isolated and immediately placed in Dulbecco’s modified Eagle’s cell culture medium (DMEM; Sigma-Aldrich Chemie, Steinheim, Germany). The urinary bladder body was first cut in half, and one half was placed on an insert with a circular opening (2 mm diameter). The insert was placed between EasyMount^®^ half-chambers of a Ussing chamber (Physiologic Instruments, San Diego, CA, USA). No pharmacological agents were used to relax the bladder smooth muscle. In the diffusion chambers, the temperature was maintained at 36–37 °C, and the incubation solutions were constantly oxygenated and stirred with gas (95% O_2_, 5% CO_2_).

During the experiments, the serosal side of the tissue was exposed to DMEM, while the urothelium was exposed to different solutions (Supp_S1). Electrophysiological parameters (potential difference and current flow) were measured using a multichannel voltage/current clamp (model VCC MC6, Physiologic Instruments, San Diego, CA, USA) as described in our previous study [51]. Electrical readings were recorded every 10 min for the first 30 min (an equilibration period), every 5 min from 30 to 60 min, every 10 min from 60 to 120 min, and every 20 min thereafter. Then, 0.01% (*w*/*v*) dispersion of poly-L-lysine hydrobromide (PLL) with a molecular weight of 30–70 kDa (Sigma-Aldrich Chemie, Steinheim, Germany) was added to the urothelial side of the chamber for 15 min after the 30 min equilibration period. The dispersion was prepared in a phosphate buffer saline (PBS) consisting of 0.94 g Na_2_HPO_4_, 0.19 g KH_2_PO_4_, and 8.00 g NaCl in 1 l of deionized water. The pH of the dispersions was adjusted to 6.5. Bladders that were not treated with PLL were exposed to PBS with a pH of 7.4.

The electrical resistance was determined according to Ohm’s law. Subsequently, the electrical resistance of the liquid without the tissue sample was subtracted, and the net electrical resistance of the urothelium was multiplied by the exposed tissue area (0.0314 cm^2^) to obtain TEER. At the end of the equilibration period, the mean absolute TEER values for the control and CYP-treated animals were 159 ± 49 Ωcm^2^ and 121 ± 67 Ωcm^2^, respectively. Since the TEER values of each bladder sample already varied considerably during the equilibration period, the results are expressed as a percentage of the TEER value of the respective tissue at the end of the equilibration period. This ensures better comparability and repeatability of the data. For the statistical analysis, the relative TEER values obtained at 45, 60, 90, 120, 180, and 360 min time points were used.

### 4.3. Determination of Potassium and Creatinine Concentration in Urine

Urine samples were collected upon animal euthanasia. The spontaneously voided urine of the animals was collected at the bottom of the euthanasia chamber and stored at −20 °C. Urine potassium (K^+^) and creatinine concentrations were measured using standard laboratory methods. Specifically, urinary K^+^ concentrations were measured potentiometrically using an electrolyte analyzer (Roche 9180; Roche, Mannheim, Germany) according to the manufacturer’s instructions. The Roche 9180 electrolyte analyzer operates on the principle of ion-selective electrode measurement for the precise determination of electrolyte concentrations. Urinary creatinine concentrations were measured using an automated biochemical analyzer (RX-Daytona+; Randox, Crumlin, UK) and a Randox Jaffe creatinine assay (Randox, Crumlin, UK) according to the manufacturer’s instructions. The obtained urine K^+^ concentrations were subsequently normalized to the urine creatinine concentrations measured in the same sample.

### 4.4. Obtainment of Urothelial Tissue Samples, mRNA Extraction, and qPCR Analysis

The halves of urinary bladders were placed in a dispersion of neutral dispase (Dispase I (neutral protease, grade I) from Sigma-Aldrich Chemie, Steinheim, Germany) with a concentration of 2 U/mL immediately after excision. The tissue was incubated in the enzyme dispersion for 30–40 min at 37 °C until the urothelium was easily separated from the underlying tissue. The urothelial samples were stored in cryovials and snap-frozen with liquid nitrogen.

Urothelial tissue samples were disrupted using TissueLyser LT (Qiagen, Hilden, Germany) and 5 mm stainless steel beads at 50 Hz (2 × 5 min). For total RNA extraction, a QIAzol Lysis Reagent and an RNeasy Plus Universal Mini Kit (both from Qiagen, Hilden, Germany) were used according to the manufacturer’s instructions. The concentration and purity of the total RNA were measured using a NanoDrop 2000 spectrophotometer (Thermo Fisher Scientific, Waltham, MA, USA).

Next, 1 µg of total RNA was transcribed into cDNA using a 1st Strand cDNA Synthesis Kit for RT-PCR (Roche, Basel, Switzerland) according to the manufacturer’s protocols. A qPCR analysis of *Cldn2* was performed using a TaqMan Gene Expression Assay (Mm00516703) and TaqMan Universal PCR Master Mix (both from Thermo Fisher Scientific, Carlsbad, SA, USA). For the other genes analyzed, 5× FIREpol HOT FIREPol EvaGreen qPCR Mix Plus (Solis Biodyne, Tartu, Estonia) and self-designed primers (Integrated DNA Technologies, Coralville, IA, USA; Supp_S2) were used. qPCR was performed on a LightCycler 480 System (Roche, Basel, Switzerland) on duplicates of each sample. Data were analyzed using the comparative Ct method relative to the expression of the endogenous control (*L32*), and they are presented as a negative Δ Ct between the average Ct of the gene of interest and the average Ct of the endogenous control.

### 4.5. RNA Sequencing Data

The mRNA transcriptome data from our previously published study [21] (available in the NCBI Gene Expression Omnibus database (https://www.ncbi.nlm.nih.gov/geo/) with the accession number GSE221783; accessed on 17 March 2023) were used. For the relevant gene sets, the information on Fragments Per Kilobase Million (FPKM) was extracted for each gene. In order to compare gene expressions between groups, fold change and adjusted *p* values (*p*_adj_) were additionally extracted. When applicable, the RNA seq data were filtered through the scRNA seq data of mouse bladder mucosa provided by Yu et al. [52]. For the visualization of the data obtained with RNA seq, ClustVis web tool [53] and SRplot (available at https://www.bioinformatics.com.cn/en; accessed on 20 June 2023) were used.

### 4.6. Immunofluorescence Labeling of Moesin and Quantitative Image Analysis

Excised urinary bladders were immediately fixed in 10% buffered formalin for 2 h at 4 °C, followed by overnight incubation in 30% saccharose at 4 °C. The bladders were embedded in tissue freezing medium (Leica Biosystems, Deer Park, IL, USA), frozen, and cut into 5 µm cryosections using a cryostat (Leica Biosystems, Deer Park, IL, USA). After drying at room temperature (RT) for 2 h, the sections were washed in PBS, permeabilized in 0.4% Triton X-100 (Sigma-Aldrich Chemie, Steinheim, Germany) for 5 min at RT, and blocked in 10% fetal bovine serum for 1 h at 37 °C. Sections were incubated overnight in a suspension of primary antibody raised against moesin (1:100; #ab52490, Abcam, Cambridge, UK) at 4 °C and the next day in the suspension of AF488-conjugated secondary antibody for 1 h at 37 °C. Sections were mounted in Vectashield with DAPI (Vector Laboratories, Burlingame, CA, USA) and examined with an LSM 900 confocal microscope (Zeiss, Jena, Germany).

For the quantification of moesin expression, the micrographs of the urothelium were taken under identical conditions in each section (63× magnification, 2% laser power for fluorescence of GFP and 0.3% laser power for fluorescence of DAPI, Airyscan detector). The micrographs (Supp_S15) were subsequently processed in Zen 3.4 (blue edition) software (Zeiss, Jena, Germany) for the determination of fluorescence intensity (FI). FI was measured only in the urothelium at 5 regions of interest (ROI) per section, and expressed as the arithmetic mean of the intensity for each ROI. The obtained FI values are expressed as a fold change relative to the FI obtained in the negative control (a tissue section immunolabeled with the omission of primary antibodies).

### 4.7. Lectin Histochemistry and Lectin Binding Evaluation

Immediately after excision, halves of the bladders were first fixed in 10% buffered formalin for 24 h, then dehydrated in ethanol series, and embedded in paraffin. The 5 µm thick paraffin sections were used for lectin histochemistry. The bladder sections were deparaffinized and rehydrated in decreasing concentrations of ethanol. After brief washing in PBS, the sections were blocked in 3% bovine serum albumin diluted in PBS for 1 h at RT. Subsequently, the sections were incubated with various FITC-conjugated lectins (Concavalin A (ConA) at 1:200, *Datura stramonium* lectin (DSL) at 1:100, Jacalin at 1:400, Wheat Germ agglutinin (WGA) at 1:800, all from Vector Labs, Burlingame, CA, USA) in PBS for 1 h at RT. Nuclei were visualized using Höechst staining (Thermo Fisher Scientific, Carlsbad, SA, USA) for 15 min at RT, and sections were mounted in Vectashield (Vector Labs, Burlingame, CA, USA).

Quantification of lectin binding was performed as previously described [35]. Briefly, lectin-labeled sections were first examined and photographed with an AxioImager.Z1 fluorescence microscope (Zeiss, Jena, Germany) at 10× magnification. All images were acquired with the same exposure time (840 ms) at an excitation wavelength of 450–490 nm for green fluorescence for all lectins. The fluorescence intensity (FI) was measured using AxioVision Rel 4.8 software (Zeiss, Jena, Germany) on at least 4 regions of interest (ROI) per section. Specifically, the urothelium was manually outlined on each ROI, and the densitometric values of the selected region were converted to gray values. The gray values obtained on individual ROI were subsequently normalized to 10,000 µm^2^ and used for quantification and statistical analysis.

### 4.8. TEM Sample Preparation for the Glycocalyx Visualization

The excised urinary bladders were immediately placed in a mixture of 4.5% paraformaldehyde and 2% glutaraldehyde in 0.2 M cacodylate buffer (pH 7.3) and cut into small pieces of tissue (1–2 mm^3^) to fix at 4 °C for 3 h. After overnight rinsing in 0.33 M sucrose in 0.2 M cacodylate buffer at 4 °C, post-fixation was performed with 1% osmium tetroxide (OsO_4_) (Carl Roth, Karlsruhe, Germany) and 0.8% potassium ferrocyanide (K_4_[Fe(CN)_6_] × 3H_2_O) (Sigma-Aldrich Chemie, Steinheim, Germany) in 0.2 M cacodylate buffer for 1 h in the dark at RT. After post-fixation, the tissue pieces were rinsed with distilled water and contrasted with 2% uranyl acetate for 1 h in the dark at RT and rinsed again with distilled water. After dehydration in an ethanol series, the tissue samples were first impregnated with propylene oxide and then left overnight in a mixture (1:1) of propylene oxide and Epon resin (Serva Electrophoresis, Heidelberg, Germany). The next day, the tissue pieces were embedded in pure Epon resin, and polymerization was carried out for five days at temperatures ranging from 35 °C to 80 °C. Semi-thin sections (1 µm) were stained with 1% toluidine blue and 2% borate in distilled water and viewed with a Nikon Eclipse TE bright-field microscope (Nikon, Amsterdam, The Netherlands). Ultrathin sections (50–60 nm) were collected on copper grids, contrasted with aqueous uranyl acetate for 20 min and lead citrate for 2 min, and examined at 80 kV with a Philips CM100 transmission electron microscope (Philips, Eindhoven, The Netherlands) equipped witha CCD camera (AMT, Woburn, MA, USA).

### 4.9. TEM Sample Preparation for the Permeability Study

For the study of the paracellular permeability properties of the urothelium, lanthanum nitrate (LaNO_3_) (Sigma-Aldrich Chemie, Steinheim, Germany) was used as a molecular tracer. The excised urinary bladders were cut in half and placed in a mixture of 2.5% glutaraldehyde and 4% paraformaldehyde with 1% LaNO_3_ in 0.1 M cacodylate buffer (pH 7.3) for 2.5 h. The LaNO_3_ in the fixative solution was then removed, and the tissue was rinsed overnight with 1% in 0.1 M cacodylate buffer. The next day, the central intact parts of the bladder halves were cut into small tissue pieces (1–2 mm^3^), which were post-fixed for 1 h with 2% osmium tetroxide (OsO_4_) (Carl Roth, Karlsruhe, Germany) dissolved in 0.5% LaNO_3_ buffered in 0.1 M cacodylate buffer. The tissue pieces were then rinsed in 0.1 M cacodylate buffer for 1 h, dehydrated in an ethanol series, and impregnated first with propylene oxide and later in a mixture (1:1) of propylene oxide and Epon resin (Serva Electrophoresis, Heidelberg, Germany). The next day, the tissue pieces were embedded in pure Epon resin, and polymerization was carried for five days at temperatures ranging from 35 °C to 80 °C. Semi-thin sections (1 µm) were prepared and stained with 1% toluidine blue and 2% borate in distilled water and subsequently viewed with a Nikon Eclipse TE bright-field microscope (Nikon, Amsterdam, The Netherlands). Ultrathin sections (50–60 nm) were collected on copper grids, contrasted with aqueous uranyl acetate for 20 min and lead citrate for 2 min, and examined at 80 kV with a Philips CM100 transmission electron microscope (Philips, Eindhoven, The Netherlands) equipped with a CCD camera (AMT, Woburn, MA, USA).

### 4.10. Estimation of Urothelial Hyperplasia by Nuclear Density

The number of urothelial nuclei per unit length, hereafter denoted as density, was estimated from the micrographs of the bladder tissue sections previously immunolabeled for Cytokeratin 7 and DAPI to respectively visualize the urothelium and its nuclei. The micrographs were obtained at 20× magnification using tan AxioImager.Z1 fluorescence microscope (Zeiss, Jena, Germany), and 5 regions of interest were analyzed for each tissue section.

The calibrated fluorescence images were exported as TIF files using AxioVision Rel 4.8 software (Zeiss, Jena, Germany). The conversion from pixels to actual lengths was performed by utilizing the provided scalebars.

The analysis was performed in three steps (Supp_S3_Figure_S1). First, the initial segmentation of both the urothelium and the nuclei therein was carried out in Ilastik (1.4) using random forest classifiers [54]. Second, the initial segmentation was improved using a combination of automatic and manual procedures in Fiji (1.53t) [55]. Finally, the density was calculated by dividing the number of detected nuclei within the segmented section of the urothelium with its length. This was conducted using a custom-built algorithm in Python (3.10.11) from Anaconda, Inc. (23.1.0) (Austin, TX, USA). A detailed description of each analysis step is available in the Supplementary Materials (Supp_S3).

The accuracy of the automated algorithm was tested by comparing its results with those obtained by manual counting on a smaller subset of images. The differences between the number of detected nuclei were minor (Supp_S3_Figure_S2). Furthermore, we performed a sensitivity analysis to assess the effect of the filtering parameters used for nuclei detection on the final results. The filtering parameters also had a negligible effect on the number of detected nuclei (Supp_S3_Figure_S3). We therefore conclude that our automated analysis is a robust and accurate method for estimating the urothelial hyperplasia. The chosen thresholds had a minimal impact on the final results, as shown by the sensitivity analysis, where we systematically inspected a range of thresholds (0 ≤ A_min_ ≤5 µm^2^, 3A_med_ < A_max_ < 5A_med_; Supp_S3_Figure_S3).

### 4.11. Statistical Analysis

A statistical analysis of the obtained data was performed using GraphPad Prism 8.0 (Dotmatics, Boston, MA, USA). The normality of the data distribution was tested using the Shapiro–Wilk test. Depending on the normality of the data distribution, statistical differences between two groups were calculated using the Mann–Whitney U test or an unpaired Student’s t test. All tests were two-tailed, and a *p* value < 0.05 was considered statistically significant.

## Data Availability

The data presented in this study are openly available in the online NCBI repository at https://www.ncbi.nlm.nih.gov/geo/ with accession number GSE221783; accessed on 17 March 2023.

## Supplementary Materials

The following supporting information can be downloaded at https://www.mdpi.com/article/10.3390/ijms242216296/s1.
