# Supplementary material for: Sex-Dependent Differences in Blood–Urine Barrier Are Subtle but Significant in Healthy and Chronically Inflamed Mouse Bladders
Source: Int J Mol Sci. 2023 Nov 14;24(22):16296. doi: 10.3390/ijms242216296 (PMC10670955; doi:10.3390/ijms242216296)
Supplement: Supplementary file 1 [file ijms-24-16296-s001.zip › ijms-2640826-supplementary.pdf]

Supplemental material*_Sex-dependent differences in blood-urine barrier are subtle but significant in healthy and chronically inflamed mouse bladders_Peskar et al.*

Supp_S1: Schematic representation of transepithelial electrical resistance (TEER) measurements. Exposure of urinary bladder wall to different solutions during the *ex vivo* experiments. Poly-L-lysine (PLL); PBS - phosphate buffer saline.

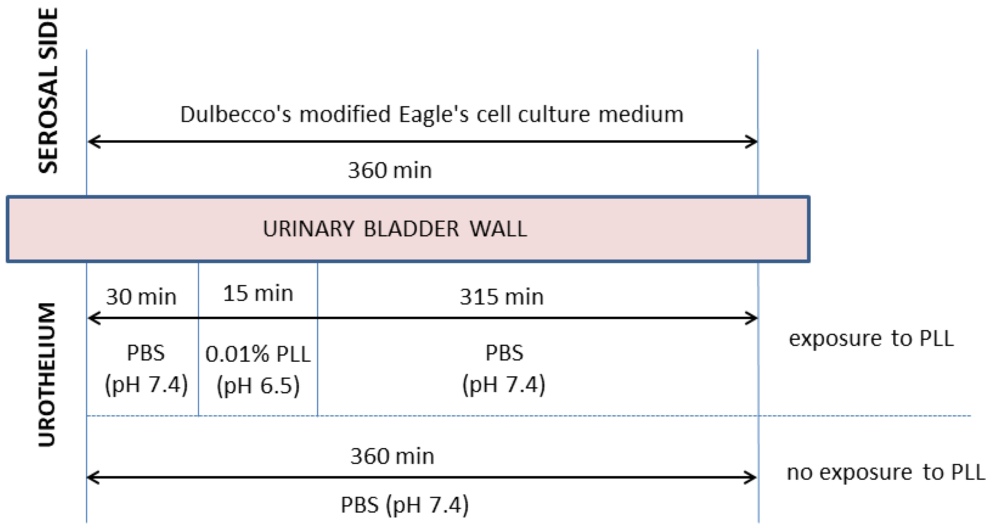

Supp_S2: List of self-designed mouse primers used for qPCR

**mRNA**	**Forward sequence (5' -> 3')**	**Reverse sequence (5' -> 3')**
L32	CCTCTGGTGAAGCCCAAGATC	TCTGGGTTTCCGCCAGTTT
Cldn1	GCCTGGAAGATGATGAGGTGC	GGCCTGGCCAAATTCATACCTGG
Cldn4	GTTCATCGTGGCAAGCATG	CCATAGGGTTGTAGAAGTCGC
Cldn5	CCAGTTAAGGCACGGGTAGC	CATAGAACTCGCGGACAACG
Cldn10	CAAGTATCAAGGCGGAGAAGG	CTTGGAAGGACTATCGTGAGG
Ctnna1	TCTCCAACACAGTCATGCCG	CAGAGTCGTCCAACTCCTCG
Ctnnb1	TGGAGGAGATAGTAGAAGGGTG	TTCAATGGGAGAATAAAGCAACTG
Gcnt3	ACCTTAACCTTCACTCCACATC	AAGTCATCTTCACAGGCGAG
Gja1	TCATTAAGTGAAAGAGAGGTGC	AAAGGCAGACTGTTCATCACC
Msn	TTCCCGTGGAGTGAAATCAG	AGATCCGCTTGTTAATCCGAAG
Tjp1	GCTTTAGCGAACAGAAGGAGC	TTCATTTTTCCGAGACTTCACCA

Supp_S3: Detailed description of nuclear density estimation workflow.

Initial Segmentation

The classifiers used for the initial segmentation were trained to identify cell nuclei and the urothelium based on a series of features at multiple scales using the pixel classification workflow in Ilastik. Training involved manually annotating (labelling) a set of images that reflected the variability in fluorescent signals within the dataset. The nucleus classifier was trained on 3 images using two labels: nuclei and the rest of the tissue including the background. The urothelium classifier was trained on 6 images by labelling the urothelium, the lamina propria (nuclei were included in both), and the background signals. The trained classifiers were then applied to the entire dataset (n = 102) to obtain the probability maps of nuclei and the urothelium. Segmentation of the urothelium proved challenging due to the non-specificity of the fluorescent dye. Therefore, these probability maps underwent a second segmentation step within Ilastik using the object classification workflow. In this step, objects detected during pixel classification were separated into different classes, namely the urothelium and the rest, utilizing multiscale features within the original images together with user-set intensity and size thresholds of the probability maps. The resulting images were exported as PNG files, manually proofread, and further processed in Fiji.

Segmentation enhancement in Fiji

The probability maps of cell nuclei were first binarized using a locally adaptive contrast thresholding (Bernsen) algorithm with a circular window (r = 10). This removed most of the noise and retained the overall masks corresponding to the nuclei. Next, holes within the masks as well as erroneous small objects (< 10 pixels) were removed using common morphological filters (fill holes, morph. open). Finally, a watershed filter was applied to separate masks that were merged due to the proximity of nuclei (Supp_3_Figure 1, top).

The urothelium segmentations were first subjected to the same morphological filters as mentioned above. However, improving these segmentations also required manual input. Each image was amended by removing detected parts of lamina propria and/or expanding the borders of isolated objects by hand. Additionally, single-pixel irregularities at the object borders were smoothed using filtered sets of Fourier descriptors (FD). Specifically, the objects were reconstructed from 4% of their FDs using the Shape Smoothing plugin (Available from: https://doi.org/10.5281/zenodo.159475) (Supp_3_Figure 1, bottom). Furthermore, the amended objects were skeletonized, and the skeletons were trimmed by removing spurious branches and short sections from their endpoints to mitigate edge effects. This was achieved using a custom-built macro within Fiji. The cleaned masks of nuclei, urothelium, and skeletons were once again manually proofread and stored as PNG files for further analysis in Python.

Calculation of (nuclear) density

First, the length of the urothelium within the micrographs was estimated by determining the length of their centrelines. This was achieved by fitting a cubic smoothing spline to each of the skeletons (goodness of fit assessed through visual inspection). Next, the isolated urothelial masks were cropped to the length of their respective skeletons. Cropping was performed perpendicular to the centrelines at their endpoints. Finally, the cropped urothelial masks were used to obtain the number and size of urothelial nuclei (Supp_3_Figure 1, right). The linear density of nuclei was calculated by dividing the number of urothelial nuclei within each urothelial mask by the length of its centrelines.

Despite our best efforts to segment individual nuclei, there was still some noise in the segmentations of the nuclei. Namely, several masks had a very large or very small surface area (A_n). The first covered multiple nuclei and could have led, despite their low number, to an underestimation of the actual nuclear density. Masks larger than a certain maximal threshold (A_{max}) were therefore counted as several smaller ones of median size (A_{med}), by rounding the quotient A_n/A_{med}. The masks with a small surface area were either *i)* cropped during isolation of the urothelial nuclei, *ii)* belonged to the nuclei that were not cut

across their largest diameter, or *iii)* were noise not removed during the filtering. This could have led to overestimation of the nuclear liner density. This was considered by removing nuclei smaller than a certain minimal threshold (A_{min}) from the analysis. The chosen thresholds had minimal impact on the final results, as shown by the sensitivity analysis, where we systematically inspected a range of thresholds (0 ≤ Amin ≤5 µm2, 3Amed < Amax < 5Amed; Supp_3_Figure 3).

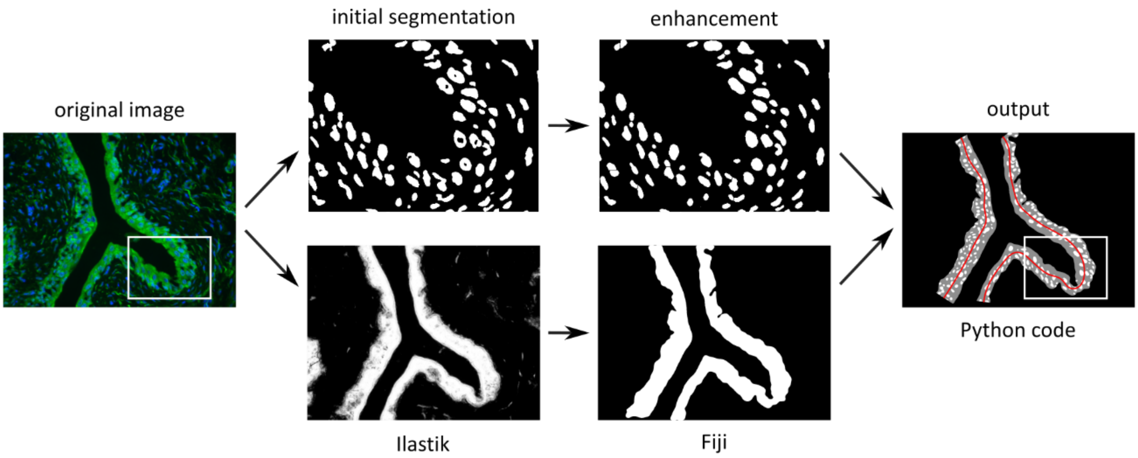

Supp_S3_Figure_S1: Analysis of fluorescent images. Raw images were used to segment the nuclei (top) and the urothelium (bottom) using Ilastik. The white rectangles indicate the region shown magnified in the example of nuclei segmentation (top middle images). Initial segmentations needed further enhancement and clean-up in Fiji. This included, but was not limited to noise removal, splitting merged objects (nuclei), isolating urothelial masks, closing holes within segmented objects. Improved segmentations were used to obtain the length of the imaged urothelium (red lines) and calculate the number of urothelial nuclei per unit length.

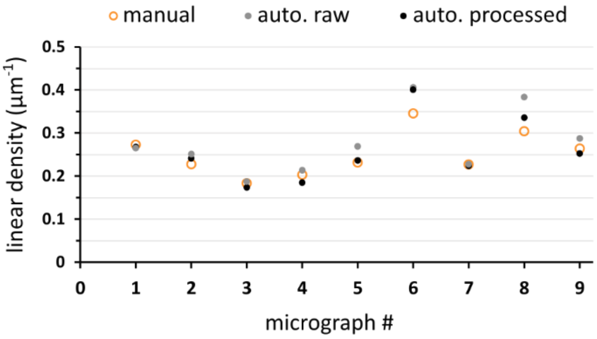

Supp_S3_Figure_S2: Automatic and manual counting yield similar results. A smaller subset of 9 images was used to assess the accuracy of the automatic counting algorithm. Manual counting results were averaged counts done by two of the authors (orange circles). Postprocessing of the automatic counts by removing nuclei smaller than 3 μm^2 and dividing the nuclei bigger than 4*the median size, brings the results close to those obtained manually (compare grey and black circles). Automatic counting performs poorly compared to manual counting only in cases with high nuclear density (> 0.35 μm^{-1}, micrograph no. 6). However, considering the majority of the data fell below 0.35 μm^{-1} this had little effect on final results.

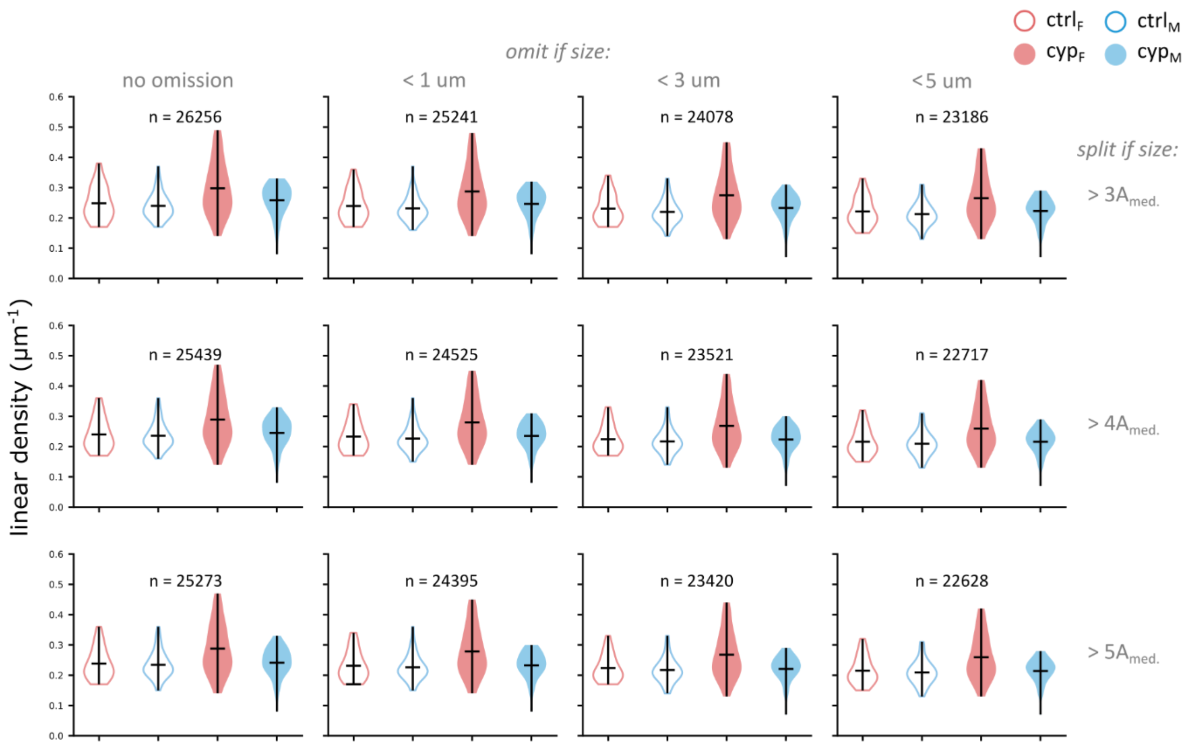

Supp_S3_Figure_S3: Postprocessing has little effect on the end results. Postprocessing was done by removal of small nuclei (from left to right) and dividing larger nuclei into several smaller ones (top to bottom). These manipulations changed the distributions of calculated linear density of the objects detected in the micrographs only slightly and did not affect the final results. The number (n) within each graph indicates the total number of detected nuclei after postprocessing. Empty and full violin plots denote controls and treated animals, respectively. Red colour denotes females, blue males. Horizontal black lines indicate the median values.

***Supplemental material**_Sex-dependent differences in blood-urine barrier are subtle but significant in healthy and chronically inflamed mouse bladders_Peskar et al.*

Supp_S4: Statistical analysis of relative TEER values. The table represents the results of statistical analysis (Mann-Whitney U test) comparing different groups at designated time points. The p-values marked in red were considered statistically significant ($p < 0.05$).

Type of TEER comparison		Time points (min)	p-value
Comparison between Ctrl and CYP animals with exposure to PLL	male animals	45	0.730
		60	0.905
		90	0.556
		120	0.413
		180	0.063
		360	0.730
	female animals	45	0.343
		60	0.200
		90	0.886
		120	0.686
		180	**0.029**
		360	**0.029**
Comparison between male and female animals with exposure to PLL	Ctrl animals	45	1.000
		60	1.000
		90	0.343
		120	1.000
		180	0.343
		360	1.000
	CYP animals	45	0.190
		60	0.063
		90	0.556
		120	0.556
		180	**0.032**
		360	**0.032**
Comparison between male and female animals without exposure to PLL	Ctrl animals	45	**0.029**
		60	**0.029**
		90	**0.029**
		120	**0.029**
		180	0.057
		360	0.200

Supp_S5: Expression profile of claudins in healthy mouse bladders. List of claudin genes was generated according to Mineta et al. [1]. The original list containing 27 genes was filtered through our RNA seq data obtained on the samples of whole bladder wall [2], and FPKM values of each gene (n=19) were extracted for Ctrl males and Ctrl females (3 animals per group). Independent clustering failed to demonstrate sex-specific differences in claudin expression. Rows and columns are clustered using correlation distance and average linkage. Lower and higher gene expression is indicated by blue and red color, respectively. Ctrl females (red, D1-3), Ctrl males (blue, B1-3).

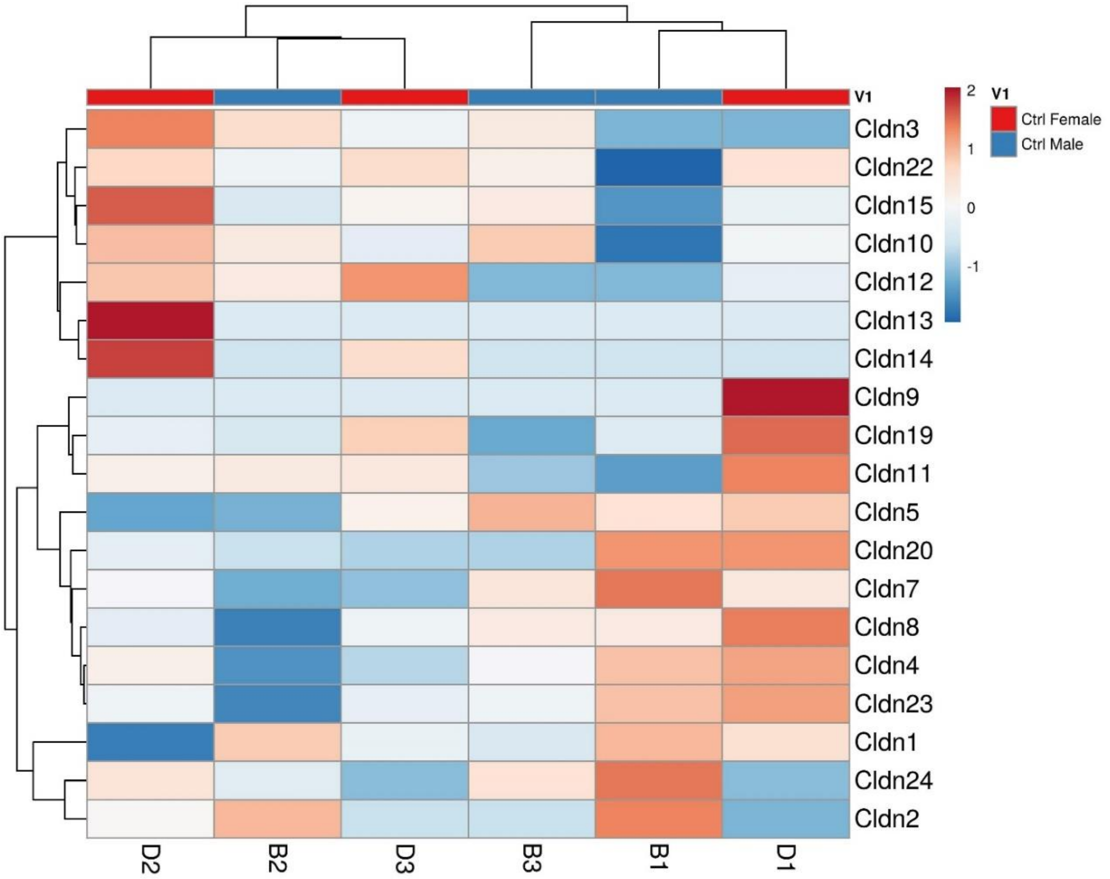

Supp_S6: Quantitative polymerase chain reaction (qPCR) validation of the expression of cell junction-related genes in urothelial tissue samples. Ctrl males, Ctrl females, CYP males, and CYP females are compared. Graphs show summary statistics of negative Δ Ct determined for 3-6 samples per group. * $p < 0.05$.

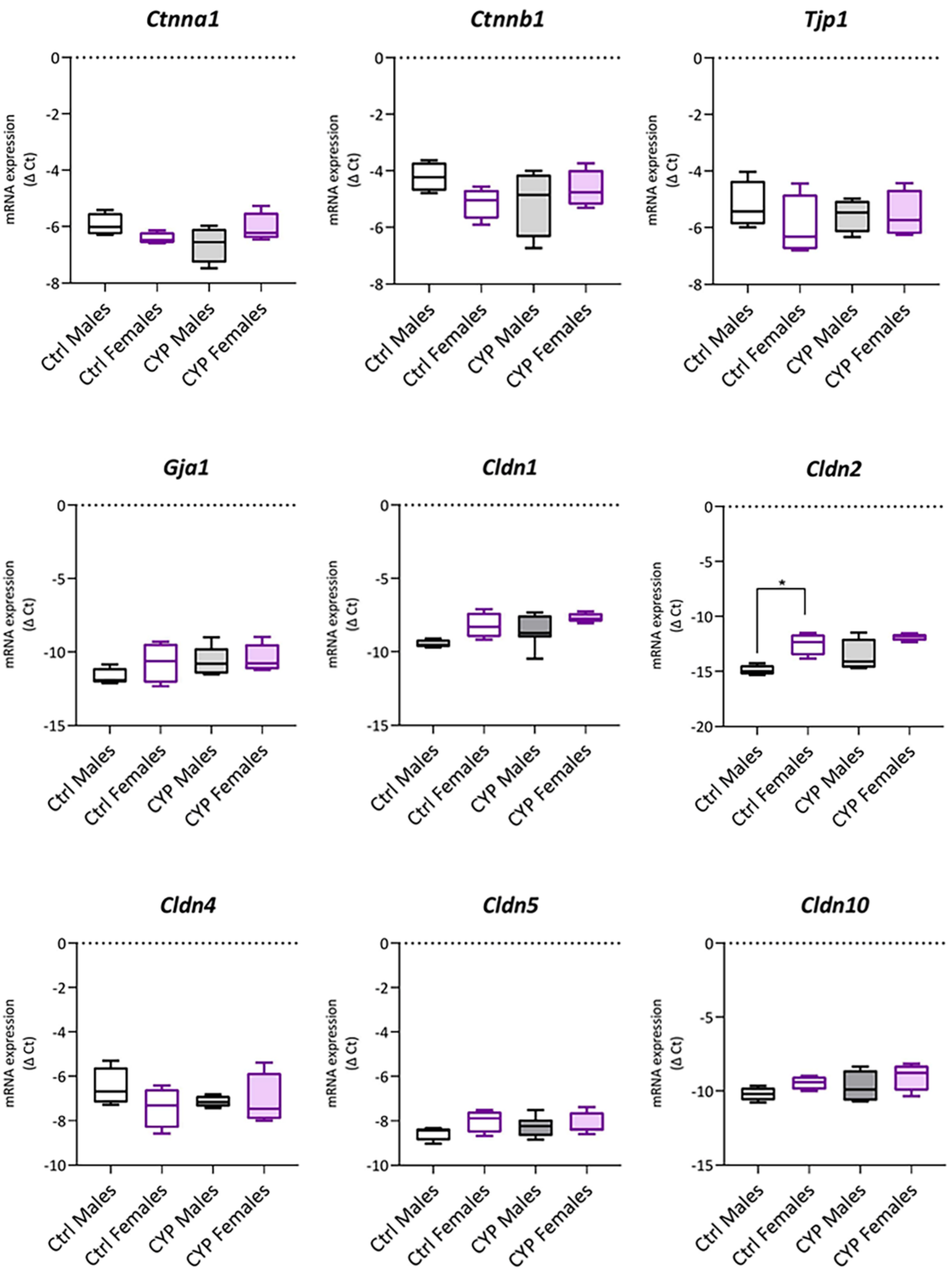

Supp_S7: Relative values of TEER measurements comparison by sex. Graphs show relative TEER values of Ctrl and CYP males (blue), and Ctrl and CYP females (red). Shaded areas represent the time frame of *ex vivo* PLL treatment of the bladder samples. Displayed are average TEER values for 2-5 animals per group (mean and SD).

The table displays the results of statistical analysis of TEER comparisons at different experimental conditions and specific time points. TEER data were not compared between CYP males without PLL and CYP females without PLL because of low number of animals included in the study.

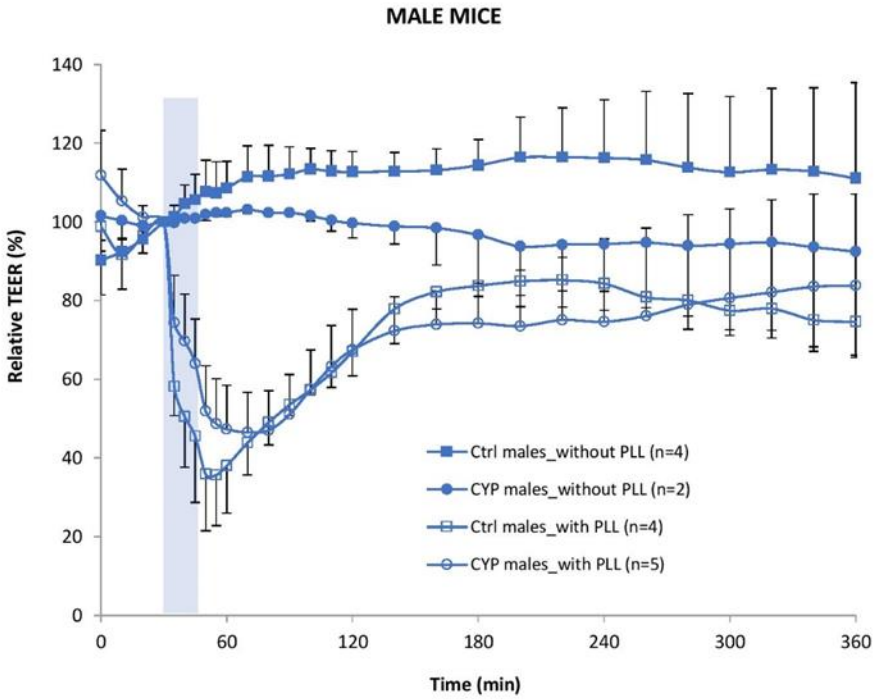

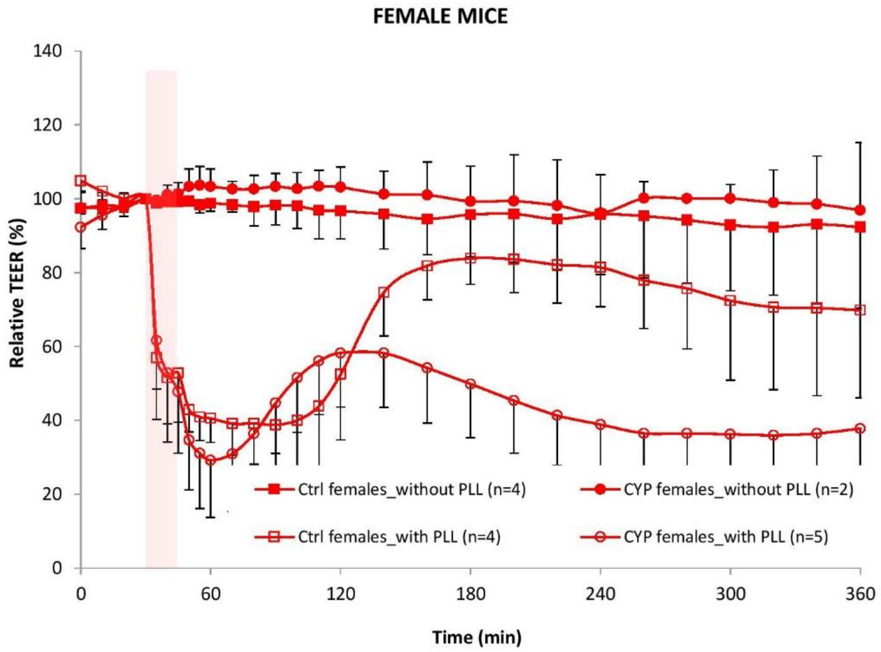

Supp_S8: Expression profile of cell junction-related genes in healthy and CYP-treated mouse bladders. Gene list was formed based on the deregulated genes (significantly and non-significantly) in males and females after CYP treatment that were determined for Tight junctions, Adherens junctions and Gap junctions pathways by KEGG analysis of RNA seq data obtained on the samples of whole bladder wall (n=63; [2]). The gene list was additionally filtered through the scRNA seq data obtained on mouse bladder mucosa [3] to increase the specificity of the data for the urothelium. Only genes with expression higher than 0 in at least one urothelial cell type remained on the list. Subsequently, data for 48 unique genes was extracted. FPKM values for Ctrl males (blue, B1-3), Ctrl females (red, D1-3), CYP males (purple, A1-3), and CYP females (green, C1-3) were compared.

Independent clustering demonstrated a significant impact of CYP treatment on the expression profile of cell junction-related genes, however no differences between sexes after treatment were detected. Rows and columns are clustered using correlation distance and average linkage. Lower and higher gene expression is indicated by blue and red color, respectively.

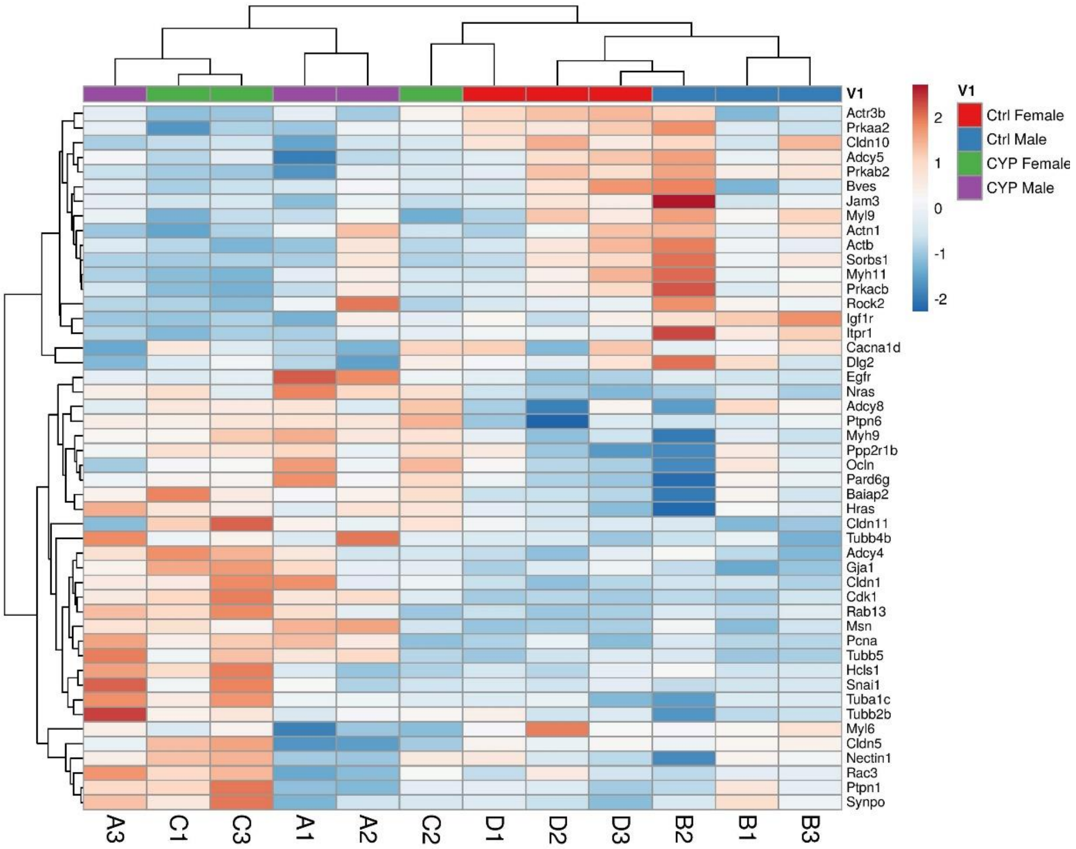

Supp_S9: Subtle differences between the sexes in the number and uniqueness of significantly deregulated genes involved in the regulation of cell junction formation. Volcano plots showing the subtle differences between sexes in the expression of genes determined for Tight junctions, Adherens junctions and Gap junctions pathways by KEGG analysis of RNA seq data obtained on the samples of whole bladder wall (n=48). Names of significantly deregulated genes ($p_{\mathrm{adj}} < 0.05$) are displayed.

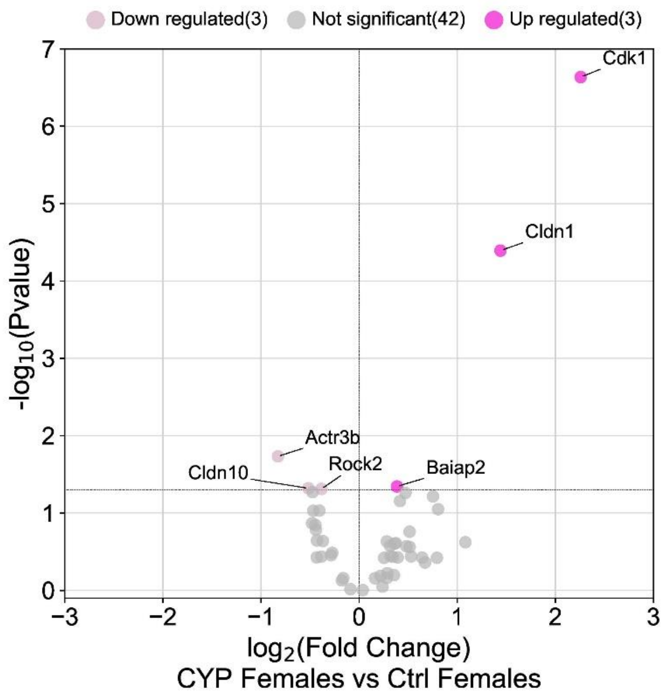

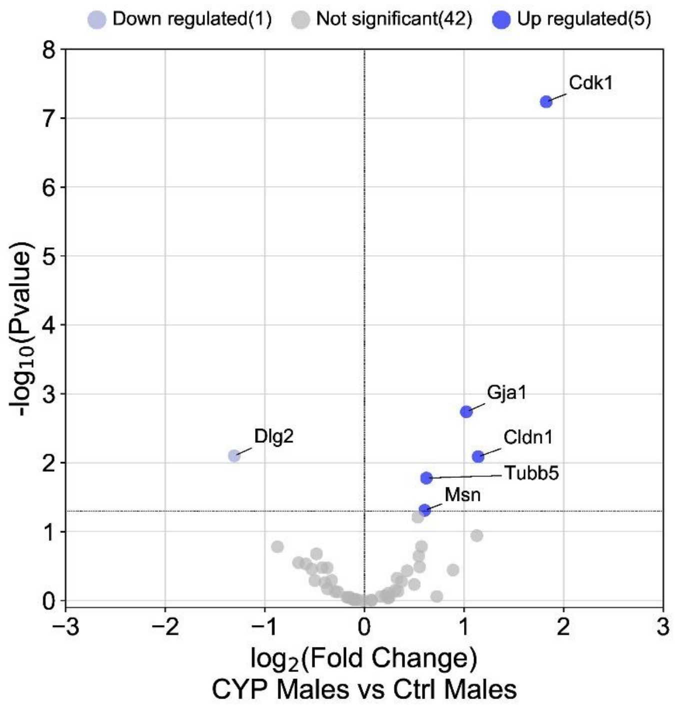

Supp_S10: Expression profile of genes involved in regulation of actin cytoskeleton in healthy and CYP-treated mouse bladders. Gene list was formed based on the deregulated genes (significantly and non-significantly) in males and females after CYP treatment that were determined for Regulation of actin cytoskeleton pathway by KEGG analysis of RNA seq data obtained on the samples of whole bladder wall (n=63; [2]). The gene list was additionally filtered through the scRNA seq data obtained on mouse bladder mucosa [3] to increase the specificity of the data for the urothelium. Only genes with expression higher than 0 in at least one urothelial cell type remained on the list. Subsequently, data for 54 unique genes was extracted. FPKM values for Ctrl males (blue, B1-3), Ctrl females (red, D1-3), CYP males (purple, A1-3), and CYP females (green, C1-3) were compared.

Independent clustering demonstrated a significant impact of CYP treatment on the expression profile of genes involved in the regulation of actin cytoskeleton, however no differences between sexes were detected. Rows and columns are clustered using correlation distance and average linkage. Lower and higher gene expression is indicated by blue and red color, respectively.

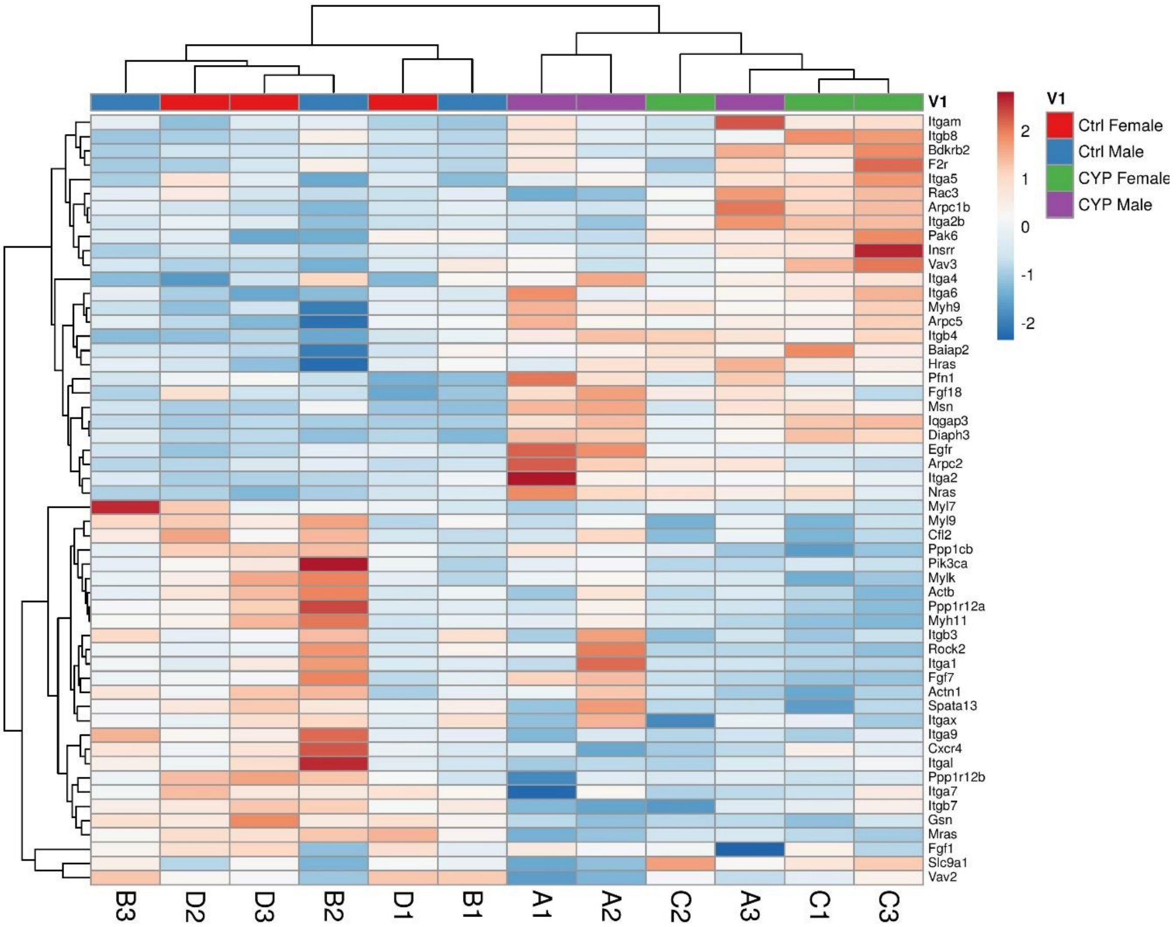

Supp_S11: Intensity of lectin binding is significantly diminished after CYP treatment in both sexes. In addition to DSL and WGA (Figure 4A and B), the binding intensity was also significantly diminished for ConA and Jacalin after CYP treatment, however no differences between the sexes were detected. The graphs show data distribution and summary statistics of grey values/10.000 µm² obtained on 4 regions of interest in 5 animals per group. * $p < 0.05$; ** $p < 0.01$; *** $p < 0.001$.

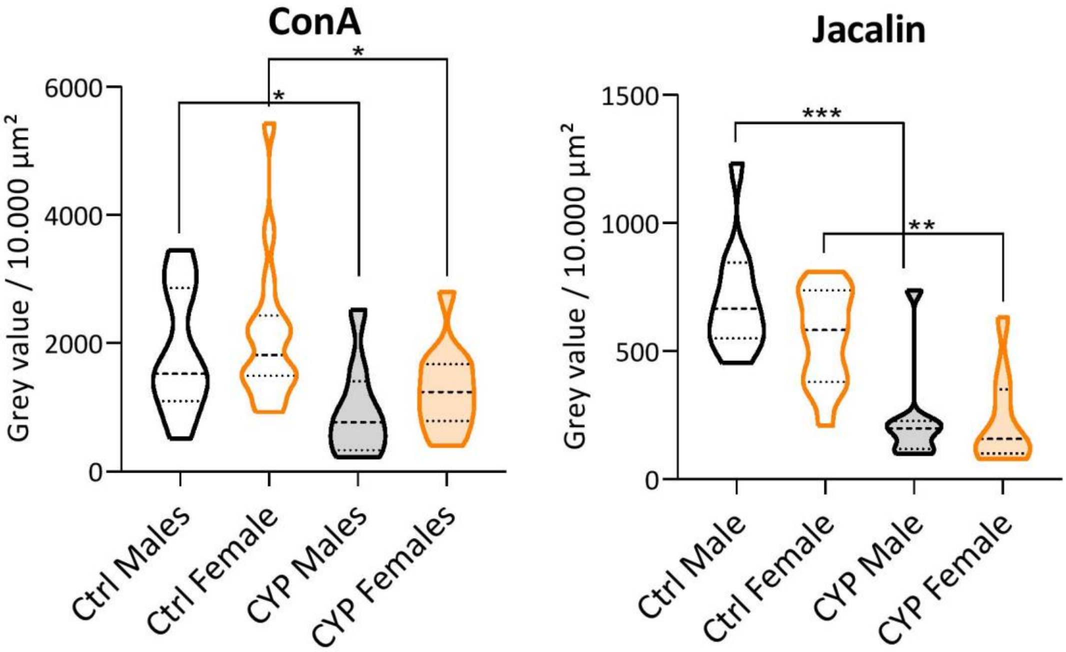

Supp_S12: Representative images of lectin histochemistry for DSL and WGA. The representative images show a reduction in lectin binding intensity to urothelium in males and females after CYP treatment (green fluorescence). Scale bars: 100 µm; dotted lines represent basal lamina; blue fluorescence: nuclei; L – bladder lumen.

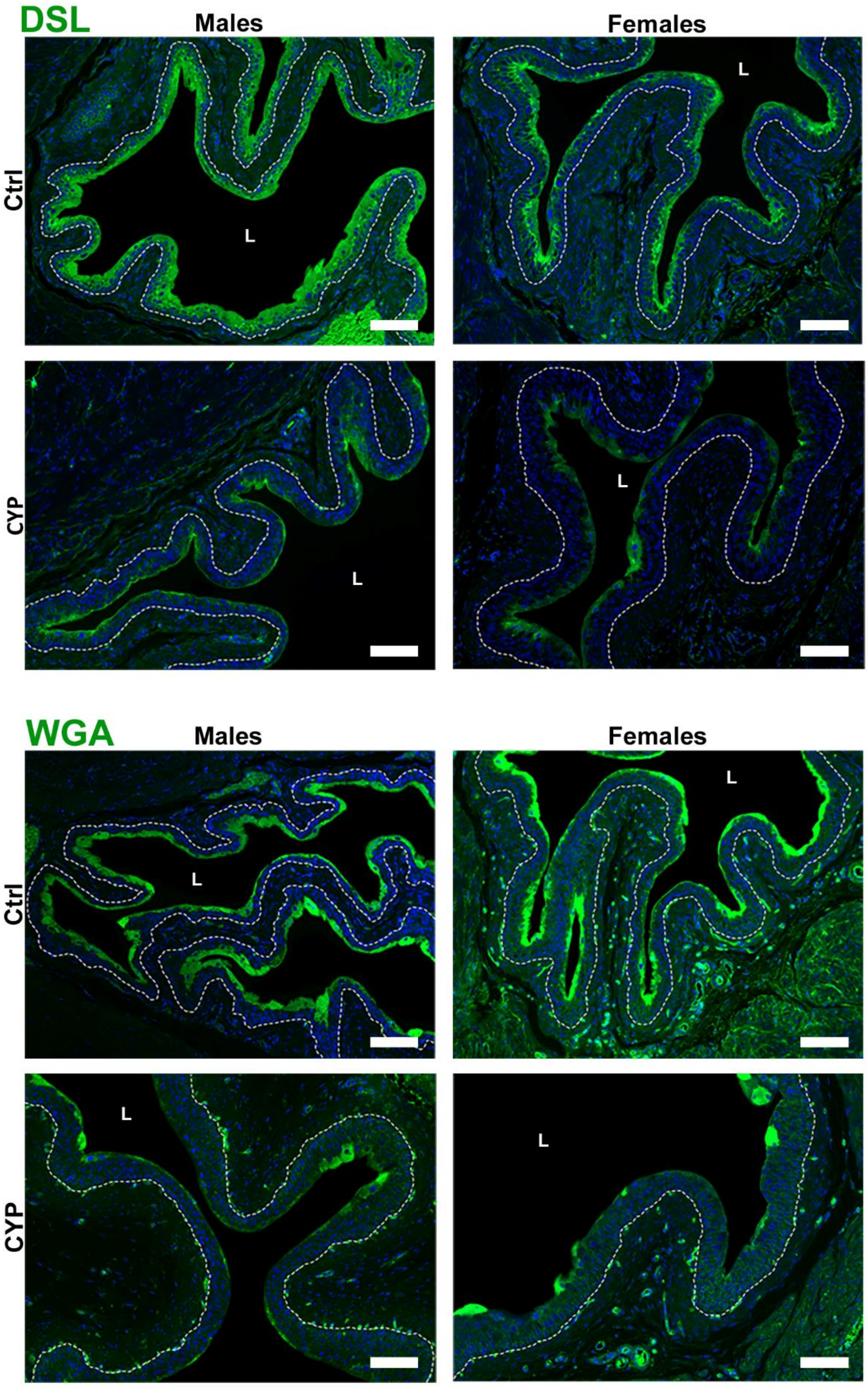

Supp_S13: List of glycosyltransferase-related genes. Search in The Human Protein Atlas (available from: https://www.proteinatlas.org/) for ''glycosyltransferase'' yielded in 251 genes, that were additionally filtered through the scRNA seq data obtained on mouse bladder mucosa [3] to increase the specificity of the data for the urothelium. Only genes with expression higher than 0 in at least one urothelial cell type remained on the list, resulting in a list of 192 unique glycosyltransferase-related genes, provided below.

Glt8d2	*Extl2*	*Alg5*	*Mgat4a*	*St3gal3*	*Stt3a*	*B3gnt8*	*Ugt1a5*
Gtdc1	*Lfng*	*B3gnt7*	*Mgat4b*	*St3gal4*	*Stt3b*	*Gxylt1*	*Ugt1a6a*
Ext1	*Mfng*	*B3gnt9*	*Mgat5*	*St3gal5*	*B3galnt1*	*Gxylt2*	*Ugt1a7c*
Plod3	*B3galnt2*	*B3gntl1*	*Ogt*	*St6gal1*	*B3galt1*	*Large2*	*Xxylt1*
Ext2	*Gyg*	*Dpagt1*	*Pofut1*	*St6galnac4*	*B3galt5*	*Mgat1*	*Xylt2*
Extl3	*Alg12*	*Fut9*	*Pomgnt2*	*St8sia1*	*B3galt6*	*Mgat3*	*A4galt*
Glt8d1	*Ddost*	*Alg2*	*Mgat2*	*St3gal2*	*St8sia6*	*B3gnt3*	*Ugt1a1*
Large1	*Alg13*	*Galnt2*	*St3gal1*	*St8sia4*	*B3gnt2*	*Piga*	*Agl*
Alg10b	*Art4*	*B4galt5*	*Csgalnact1*	*Fut2*	*Galnt15*	*Galntl6*	*Has3*
Alg11	*Art5*	*B4galt6*	*Dad1*	*Sec1*	*Galnt16*	*Gba*	*Hprt*
Alg1	*B3galt4*	*B4galt7*	*Dpm1*	*Fut4*	*Galnt17*	*Gba2*	*Mtap*
Alg14	*B3glct*	*B4gat1*	*Dpy19l1*	*Fut7*	*Galnt18*	*Gbe1*	*Nampt*
Alg3	*B3gnt5*	*C1galt1*	*Dpy19l3*	*Fut8*	*Galnt3*	*Gcnt1*	*Naprt*
Alg6	*B4galnt1*	*Cercam*	*Dpy19l4*	*Galnt1*	*Galnt4*	*Gcnt2*	*Ost4*
Alg8	*B4galt1*	*Chpf*	*Eogt*	*Galnt10*	*Galnt5*	*Gcnt3*	*Parp1*
Alg9	*B4galt2*	*Chpf2*	*Fktn*	*Galnt11*	*Galnt6*	*Gys1*	*Parp10*
Aprt	*B4galt3*	*Chsy1*	*Fut10*	*Galnt12*	*Galnt7*	*Has1*	*Parp11*
Art3	*B4galt4*	*Colgalt1*	*Fut11*	*Galnt14*	*Galnt10*	*Has2*	*Parp12*
Parp14	*Pigv*	*Pomt2*	*Sirt4*	*Uggt1*	*Parp6*	*Poglut1*	*Qtrt1*
Parp16	*Pigz*	*Ppat*	*Sirt6*	*Uggt2*	*Parp8*	*Poglut2*	*Rfng*
Parp2	*Pnp*	*Pygb*	*St3gal6*	*Umps*	*Parp9*	*Poglut3*	*Rpn1*
Parp3	*Pofut2*	*Pygl*	*St6galnac2*	*Upp1*	*Pigb*	*Pomgnt1*	*Rpn2*
Parp4	*Poc1b*	*Pygm*	*St6galnac3*	*Upp2*	*Pigm*	*Pomt1*	*Rxylt1*
Tiparp	*Tnks*	*Tnks2*	*St6galnac6*	*Xylt1*	*Slc35b4*	*Slc35d2*	*Ugcg*

Supp_S14: CYP treatment has no impact on the expression of glycosyltransferase genes in mouse bladders. FPKM values of 192 glycosyltransferase-related genes were extracted from RNA seq data obtained on the samples of whole bladder wall [2]. Gene expression profiles between Ctrl males (blue, B1-3), Ctrl females (red, D1-3), CYP males (purple, A1-3), and CYP females (green, C1-3) were compared.

Independent clustering failed to demonstrate a significant impact of CYP treatment on the expression of glycosyltransferase-related genes. Rows and columns are clustered using correlation distance and average linkage. Lower and higher gene expression is indicated by blue and red color, respectively.

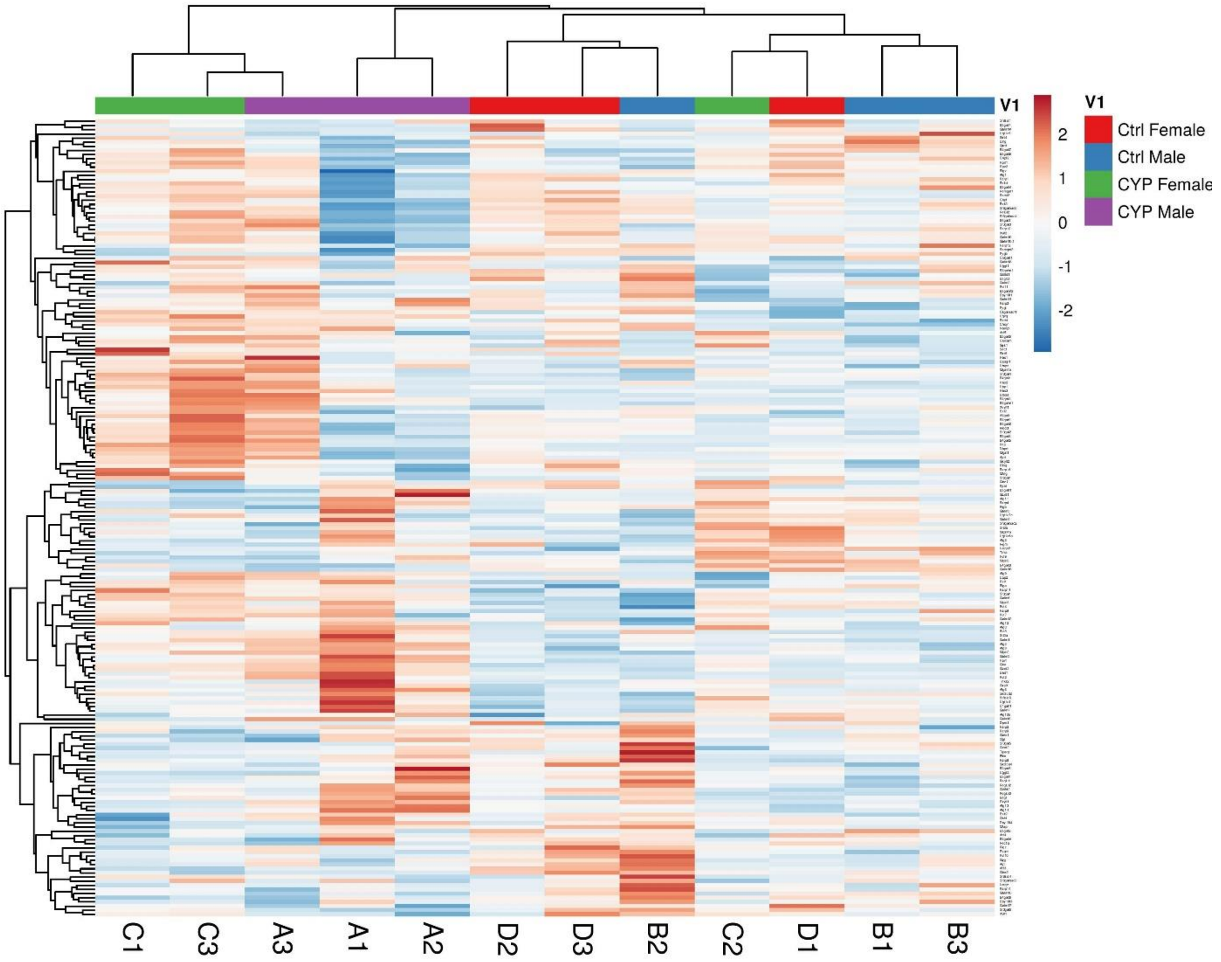

Supp_S15: Representative micrographs of moesin expression in the urothelium of Ctrl (A) and CYP animals (B) taken with confocal microscopy. Presented are micrographs used for measurements of fluorescence intensity. The intensity of immunolabeling of moesin (green fluorescence) in the urothelium was relatively weak compared to the endothelium of the capillaries (arrows). Nuclei are stained with DAPI (blue fluorescence). Dotted line - basal lamina; L – lumen of the urinary bladder; scale bars: 10 µm.

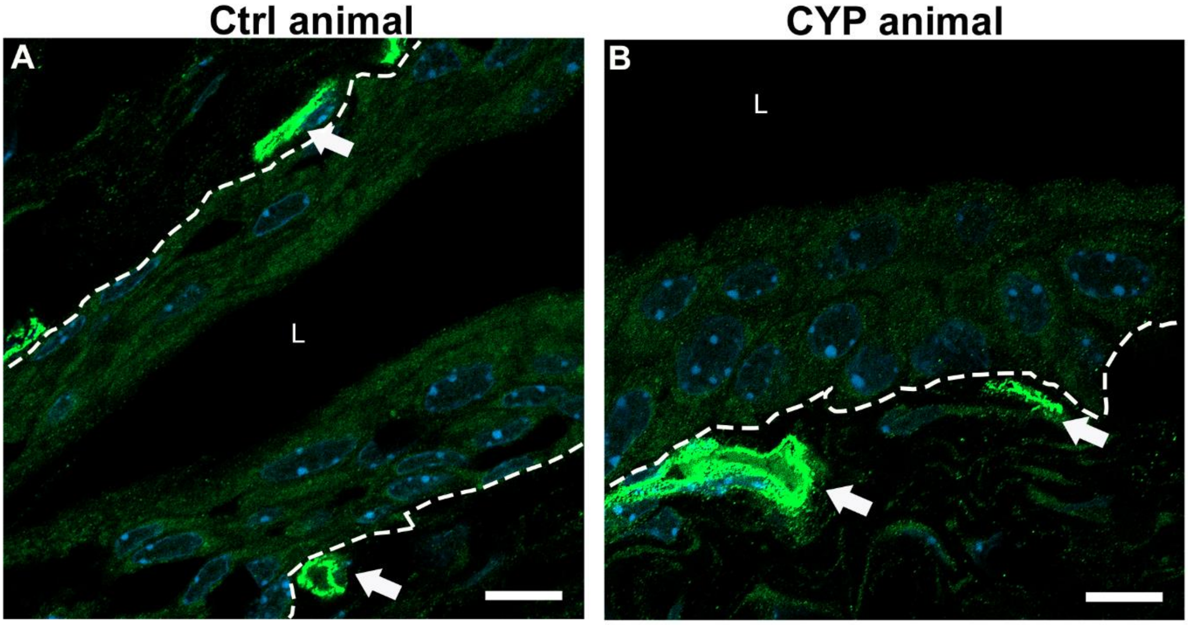

References

1. Mineta, K.; Yamamoto, Y.; Yamazaki, Y.; Tanaka, H.; Tada, Y.; Saito, K.; Tamura, A.; Igarashi, M.; Endo, T.; Takeuchi, K., et al. Predicted expansion of the claudin multigene family. *FEBS Lett* **2011**, *585*, 606-612, doi:10.1016/j.febslet.2011.01.028.
2. Peskar, D.; Kuret, T.; Lakota, K.; Erman, A. Molecular Profiling of Inflammatory Processes in a Mouse Model of IC/BPS: From the Complete Transcriptome to Major Sex-Related Histological Features of the Urinary Bladder. *Int J Mol Sci* **2023**, *24*, doi:10.3390/ijms24065758.
3. Yu, Z.; Liao, J.; Chen, Y.; Zou, C.; Zhang, H.; Cheng, J.; Liu, D.; Li, T.; Zhang, Q.; Li, J., et al. Single-Cell Transcriptomic Map of the Human and Mouse Bladders. *J Am Soc Nephrol* **2019**, *30*, 2159-2176, doi:10.1681/asn.2019040335.
